# Disproportionate exposure to extreme floods in historically redlined U.S. communities

**DOI:** 10.1038/s41467-026-76024-2

**Published:** 2026-07-23

**Authors:** Elizabeth Appel, S. Amin Enderami, Elaina J. Sutley, Admin Husic

**Affiliations:** 1 Department of Civil, Environmental and Architectural Engineering, University of Kansas, Lawrence, KS USA; 2 Department of Engineering, University of Cambridge, Cambridge, UK; 3 Department of Civil and Environmental Engineering, Virginia Tech, Blacksburg, VA USA

**Keywords:** Hydrology, Water resources

## Abstract

Extreme floods and historical public policies both leave lasting impacts. We analyzed flood exposure across the neighborhoods of 202 US cities historically assessed for lending risk by the Home Owners’ Loan Corporation on a scale from ‘best (A) to ‘hazardous’ (D). Using high-resolution flood modeling and dasymetric population mapping, we found that D-graded neighborhoods bear a disproportionate flood burden in roughly three-quarters of cities. This inequality follows a systematic gradient: A- and B-graded neighborhoods are underrepresented in the flooded population, C-graded neighborhoods are proportionately represented, and D-graded neighborhoods are overrepresented. A greater fraction of D-graded residents are exposed to the 100-yr flood (6.4%) than A-graded residents to the 1000-yr flood (5.9%) with the gap widening at higher-magnitude floods. Flood disparities are most pronounced in the Midwest and Mid-Atlantic regions, aligning with the historical concentration of industrial labor and redlined neighborhoods along waterways. However, we find that proximity to waterways alone does not explain the observed inequality: flood disparities persist even among neighborhoods far from major rivers and coasts, implicating infrastructure deficits as a compounding factor. Neighborhoods marked as ‘hazardous’ nearly a century ago remain disproportionately vulnerable today, underscoring the need for equitable urban planning.

## Abstract (web summary)

This study examines the modern intersection of historical redlining and urban flood risk. The results show that historically redlined neighborhoods bear a disproportionate flood burden that worsens as flood severity increases.

## Introduction

Flooding is one of the most devastating and costly natural disasters. In the last decade, annual economic flood losses have been extensive, exceeding US$ 31 billion in the US^1^ and US$ 388 billion globally^2^. Extreme floods also cause a substantial loss of life each year^3^. Flood frequency and intensity are anticipated to increase in many regions under the combined effects of climate and land use change^4–7^. While considerable work has been done to estimate the human and economic costs of historic and future flooding^8–10^, the role of government-sponsored policies in potentially exposing vulnerable groups to disproportionate flood burdens is less understood.

Flooding is driven by a combination of geologic, climatic, hydrologic, and anthropogenic factors. The size of flood events can be categorized by the mean return interval (MRI)^9^, where flood magnitude is directly related to the probability of a storm occurring in a given time period (e.g., a 100-yr flood has a 1% annual probability). Recent advancements in flood modeling, such as Fathom-US^11^, now provide near-complete coverage of fluvial, pluvial, and coastal flooding extents in the US at a high spatial resolution for multiple MRIs (for example, the 100-yr and 1000-yr floods)^12^. These modeling products provide considerable improvement in spatial resolution (10-m, complete coverage of the US) over traditional maps created and used by the Federal Emergency Management Agency (FEMA) (varied local resolution, 60% coverage of the US)^12–14^. As one example, Fathom-US estimates show that the total US population exposed to flooding from a 100-yr event amounts to 41 million, which is 3.1 times greater than that predicted by the FEMA methodology^6^. There remains potential to integrate these high-resolution flood products to improve the assessment of flood disparities among the socially vulnerable.

Vulnerable populations—often comprising those below the poverty line, people of color, individuals with disabilities, and those facing housing insecurity^15^—often struggle to absorb the shocks associated with flooding due to constrained financial capital and systemic barriers to disaster recovery resources^10,16,17^. Due to economic constraints that limit residential mobility and investment in climate risk retrofits^18^, these populations are disproportionately situated in flood-prone areas^16,19^, thereby enduring greater flood risk^20–22^. To quantify the number of people in a population affected by a given hazard, traditional assessments typically rely on aggregated US Census data. The most granular census data, the block, is still quite coarse, as the mean block size is ~1 km^2^. New dasymetric population data products—that is, estimates of population that are disaggregated from coarse (1 km) to fine (30 m) resolutions^23^—are enabling a more precise analysis of how flood hazards intersect with specific communities.

Flood risk exposure, which materializes at the intersection of hazards and vulnerabilities, is shaped by geographical location, political institutions, economic interests, and community trust in decision-makers^24–27^. ‘Redlining’ is one such institution that has been investigated as a driver of unequal environmental risk exposure^18,28–30^. In the 1930s, a US government-sponsored agency known as the Home Owners’ Loan Corporation (HOLC) assigned grades to neighborhoods with the stated intention of assessing mortgage risk and expanding home buying opportunities^31^. These maps circulated across the country and codified and reinforced prevailing appraisal and lending patterns^30^. The color-coded HOLC grades include A (‘best’ in green), B (‘still desirable’ in blue), C (‘definitely declining’ in yellow), and D (‘hazardous’ in red, hence the term ‘redlining’). The HOLC grades were guided by conceptions of value that were deeply tied to race, immigration status, and income^30^, further entrenching residential segregation and limiting upward economic mobility^32^. The socioeconomic consequences of HOLC-redlining have been extensively studied^32–34^, but the links between historical HOLC-redlining and present-day flooding have only recently been explored. These recent works have focused on differential flood exposure on property across HOLC-defined borders^18^ and the lack of flood prevention infrastructure in HOLC-redlined communities^35^. However, there remains a critical need to further quantify the scale of this flood inequality as borne by people, rather than property, to understand if observed disparities can be attributed to geographic proximity to flood sources or reflect broader systemic factors, and to understand whether flood inequities grow as flood intensities increase.

The objective of this study is to investigate the relationship between historic HOLC grading of neighborhoods and modern-day flood exposure across 202 US cities. Specifically, we seek to answer: (1) does neighborhood HOLC grade play a statistically significant role in local flood exposure at the city-scale? (2) does geographic proximity to waterways fully account for these disparities? and (3) does disparity in flood exposure amplify with flood magnitude? To test the null hypothesis that no relationship exists between HOLC-grading and modern-day urban flood exposure, we leverage digitized HOLC maps, dasymetric population data, and modern high-resolution flood simulations for multiple MRIs, ranging from 100-yr to 1000-yr events. This work aims to characterize the enduring association between HOLC-graded neighborhoods and environmental inequality, examining whether the spatial patterns codified nearly a century ago correspond to present-day flood exposure disparities.

## Results

### Unequal representation in urban flooding

**Fig. 1:**
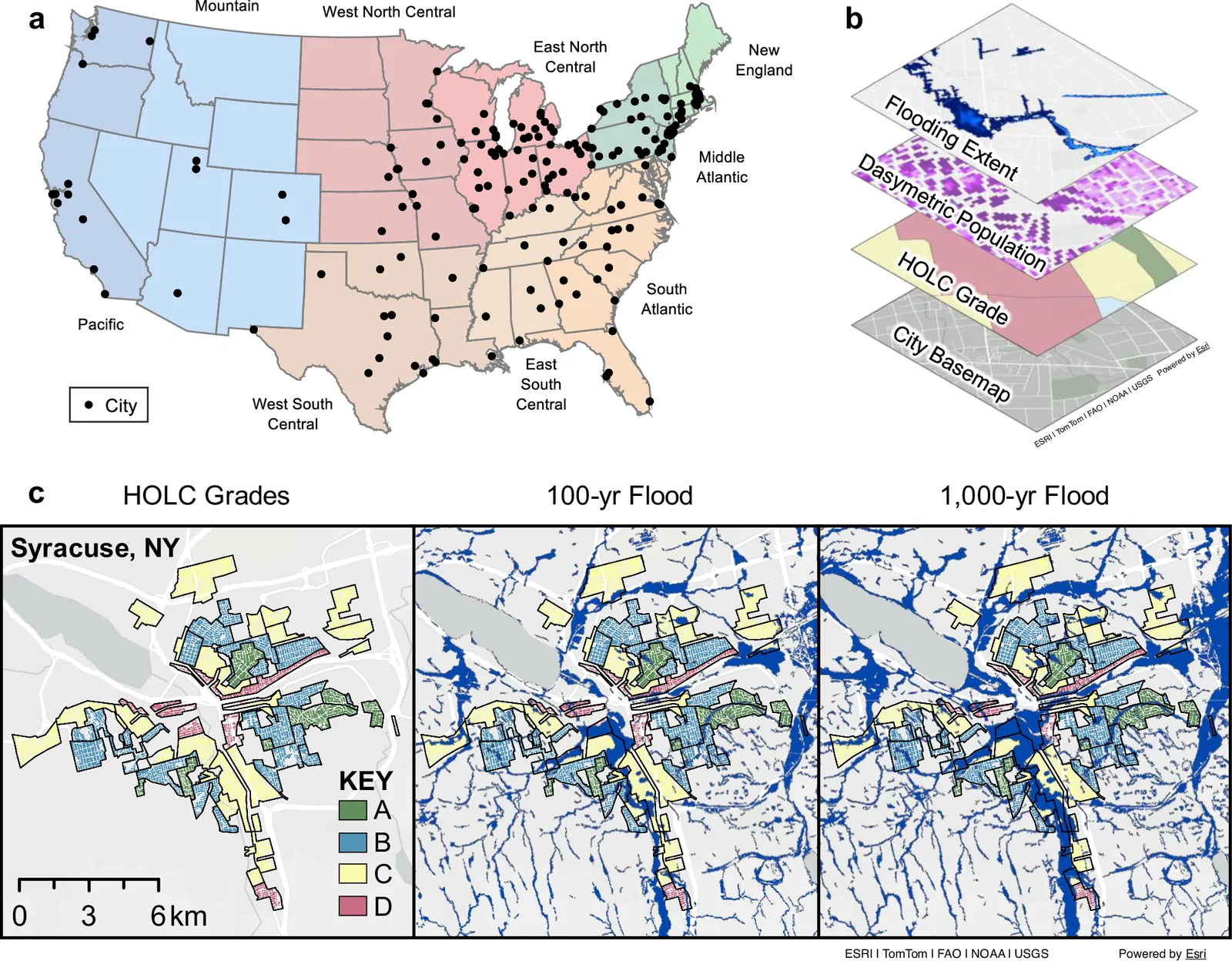
Study framework, city locations, and localized flood inundation mapping. a A map of the 202 cities in the Not Even Past redlining database and the nine U.S. Census divisions. **b** A schematic of the study concept is shown, indicating the intersection of flooding extent, population density, and Home Owners’ Loan Corporation (HOLC) neighborhood grading. **c** Flood inundation distributions for Syracuse, NY, for 100-yr and 1000-yr return intervals. Syracuse (43.0° N, 76.1° W) is in the Middle Atlantic Census division. North is oriented toward the top of the figure. HOLC grades: A – ‘best’ (green), B – ‘still desirable’ (blue), C – ‘definitely declining’ (yellow), and D – ‘hazardous’ (red). The basemaps used in (**b**) and (**c**) are the intellectual property of Esri and are used herein under license. Copyright © 2025 Esri and its licensors. All rights reserved.

Figure 1 illustrates our (a) study extent, (b) method concept, and (c) model outputs. Figure 1a shows the 202 cities in our analysis and the nine US Census divisions, which are used to assess spatial variation in flood inequality. Figure 1b provides a concept for how the data layers are used to assess flood exposure for dasymetrically populated cells within graded neighborhoods of a city. Figure 1c demonstrates the outputs of our modeling approach for a case study city: Syracuse, NY.

Syracuse was selected as a case study because it contains all four HOLC grades, it is small enough to be shown in a clear figure, and it experiences inundation patterns that mirror most of the cities studied in this paper. Specifically, Syracuse experiences flood inequality. A, B, C, and D-neighborhoods make up 9, 36, 43, and 10% of the city’s population but 5, 15, 57, and 22% of the population impacted by the 100-yr flood. A and B-neighborhoods are underrepresented in flooding, whereas C and D-neighborhoods are overrepresented.

**Fig. 2:**
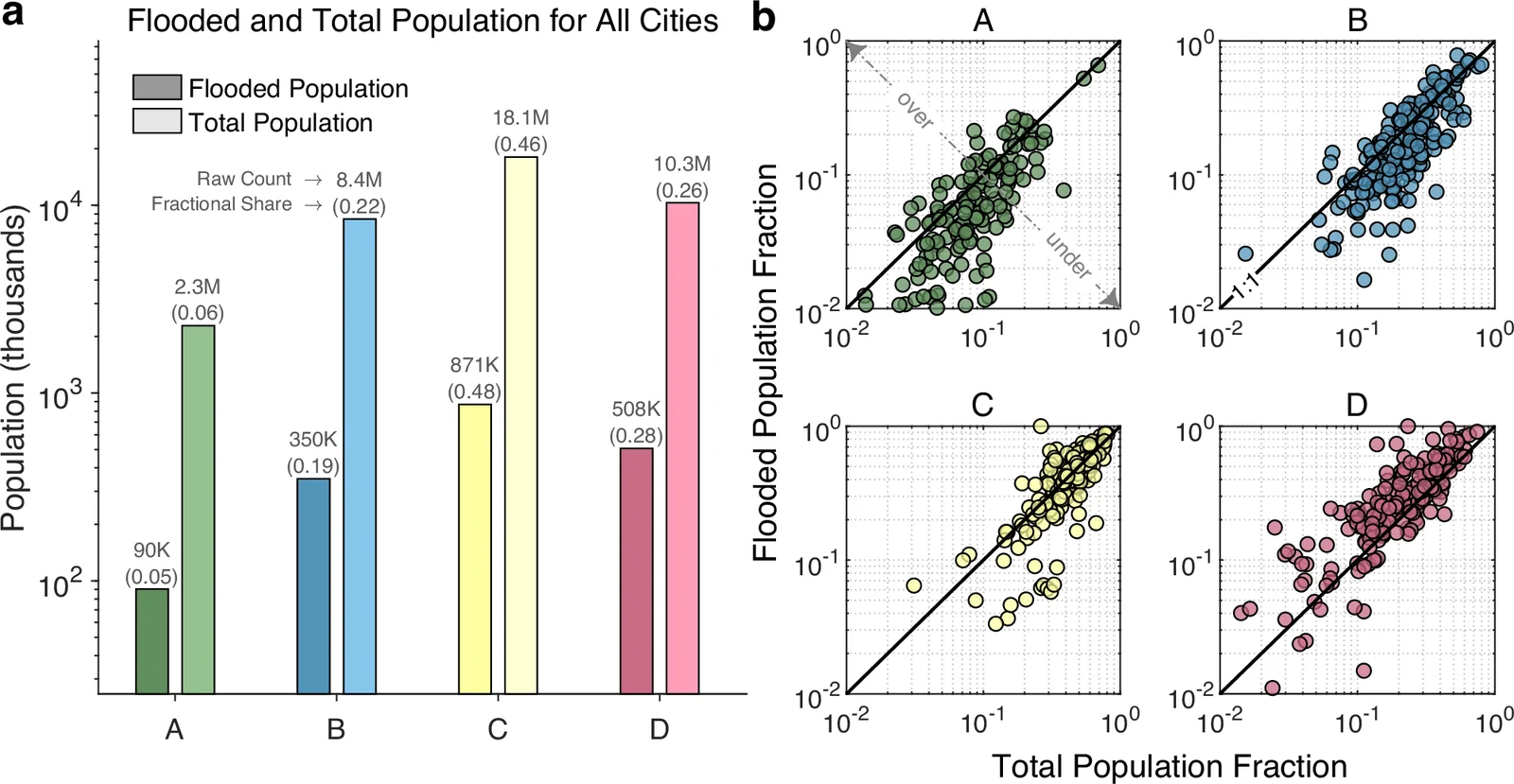
National population counts and individual city disparities in flood exposure. **a** Bars show the cumulative total population (lighter bars) and 100-year flooded population (darker bars) for all cities in the analysis by HOLC grade. Text above each bar denotes the raw population count and the fractional share of the total overall population or flooded population, respectively. **b** Markers show the 100-yr flooded population versus total population fractions for individual cities. Each panel represents a different HOLC grade. The solid black line represents a 1:1 slope, or equal share of the flood burden. Neighborhoods falling above the 1:1 line are overrepresented in flooding exposure while neighborhoods below the line are underrepresented. This figure was created with MATLAB.

Aggregated across all 202 cities, D-graded neighborhoods contain 26.4% of the total HOLC-graded population but account for 27.9% of the 100-yr flooded population (Fig. 2a). By contrast, A-graded neighborhoods represent 5.8% of the population but only 5.0% of the flooded population. C-graded neighborhoods, which house the largest share of the HOLC-graded population (46.2%), also bear the largest absolute flood burden (47.9%). Because these aggregate patterns may be skewed by a few large cities, we conduct a city-level analysis to determine if each grade’s share of the flood burden is systematically disproportionate.

We extend the flood inequality approach to all cities and investigate whether each neighborhood grade (A, B, C, and D) has an equal fractional share of the flooded population in a city (f_flood-pop_) compared to its fractional share of the total population (f_total-pop_). Our null hypothesis assumes an equal urban flooding scenario (i.e., f_flood-pop_/f_total-pop_ = 1) where HOLC-graded neighborhoods have an equal chance (50%) of being overrepresented (f_flood-pop_/f_total-pop_ > 1) or underrepresented (f_flood-pop_/f_total-pop_ < 1) in sharing the flood burden.

At the city-scale, results lead us to reject the hypothesis of an equal flood burden for all but C-graded neighborhoods (Fig. 2b). Results are shown for the 100-yr flood. For A-graded neighborhoods, 149 of 185 cities (81%) plot below the 1:1 line and a binomial sign test rejects the null hypothesis of equal flood distribution (*p* = 1.6 × 10^−17^). A similar pattern was observed for B-graded neighborhoods, with 147 of 199 cities (74%) plotting below the line of equal burden (*p* = 1.1 × 10^−11^). For C-graded neighborhoods, the proportion of cities below parity (104 of 201, 52%) is not significantly different from 50% (*p* = 0.67). D-Graded neighborhoods are significantly overrepresented in the flood burden, with 147 of 194 cities (76%) plotting above the 1:1 line (*p* = 3.5×10^−13^). These results demonstrate that C-graded neighborhoods experience flood exposure commensurate with their share of the population, A- and B-graded neighborhoods are underrepresented, and D-graded neighborhoods are overrepresented. Note that the slight overrepresentation of C-graded neighborhoods at the aggregate level in Fig. 2a reflects the outsized influence of a few large cities, such as New York City and Los Angeles, rather than a systematic national trend.

### Spatial and regional disparities in flood exposure

To assess spatial gradients in flood exposure, we describe flood inequality using a pairwise measure of flood parity defined as f_D_ – f_A_, where f is the fraction of the total population that is flooded for a given HOLC grade in a city. Perfect flood parity, that is, an equal share of the relative flood burden by D and A neighborhoods, would be given as f_D_ – f_A_ = 0. For the purposes of this study, our pairwise comparison is limited to A- and D-graded neighborhoods as they represent the opposite ends of the HOLC grading spectrum. For Syracuse, f_D_ – f_A_ is equal to 0.14 and 0.20 for the 100-yr and 1000-yr events, respectively, demonstrating that flood inequality grows with flood magnitude at the city level. While a substantial fraction of US cities follows this pattern, it is not universal (see Figs. S1, 2 for examples).

**Fig. 3:**
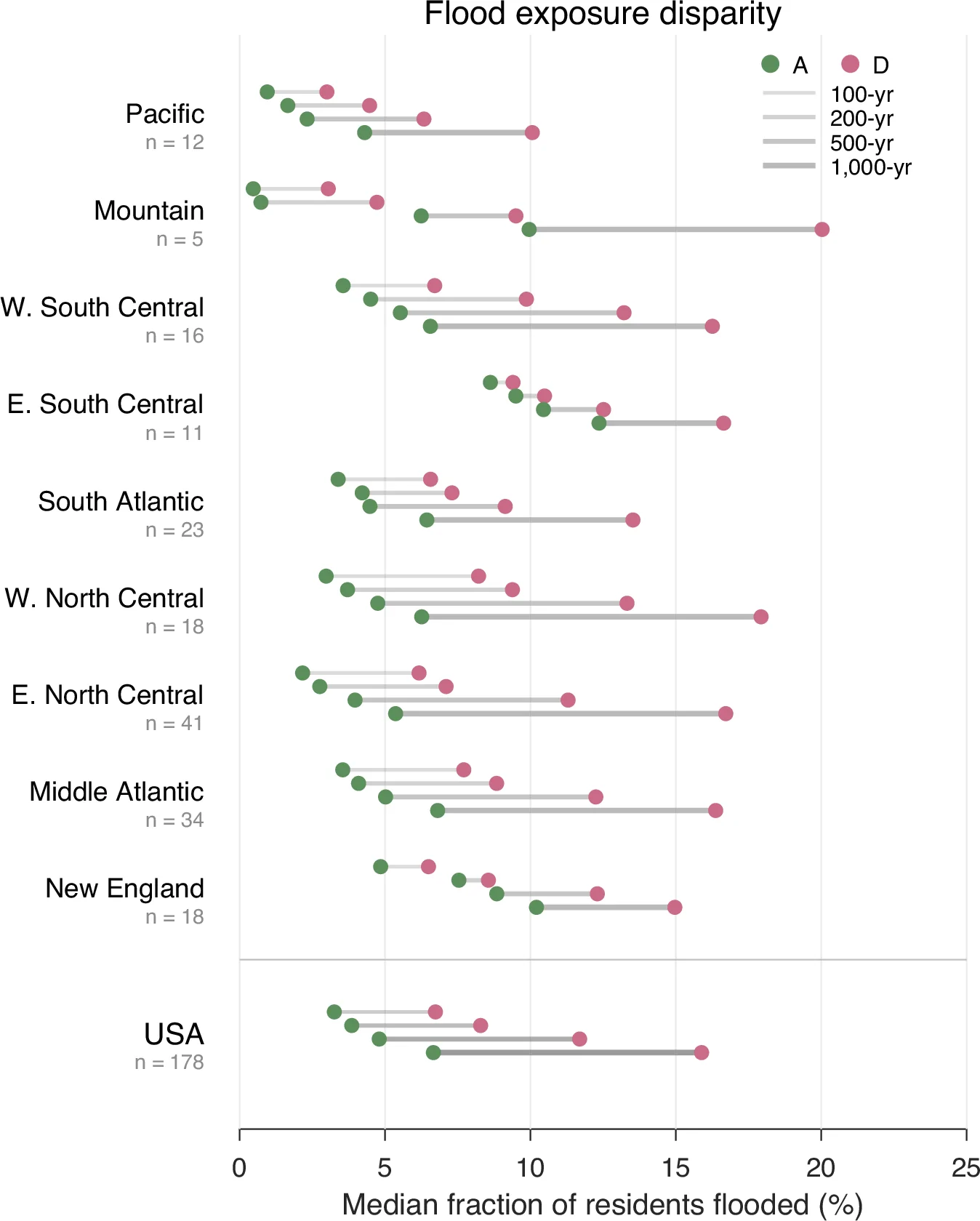
Regional and national flood exposure disparities between A- and D-graded neighborhoods. The dumbbell chart displays the median fraction of residents flooded within A-graded (green markers) and D-graded (red markers) areas. The horizontal lines connecting the markers illustrate the exposure gap between the two grades. For each geographic division and the overall national median (USA), four flood mean return intervals (MRIs) are plotted vertically from top to bottom: 100-yr, 200-yr, 500-yr, and 1000-yr. The number of cities analyzed within each region is denoted by n. This figure was created with MATLAB.

At the national scale, for the 100-yr event, we find flood disparity (f_D_ ≠ f_A_; *p* < 0.0005), with D-graded neighborhoods receiving a relatively greater share of the flood burden than A-graded neighborhoods (USA; Fig. 3). The disproportionate flooding of D-graded neighborhoods (f_D_ > f_A_) is statistically significant in the Pacific, West South Central, South Atlantic, West North Central, East North Central, and Middle Atlantic divisions. While there are 32 cities where f_A_ > f_D_, this trend is not significant for any region. Notably, the exposure gap between A- and D-graded neighborhoods widens visibly at higher MRIs (Fig. 3), with the gap lengthening from the 100-yr to the 1000-yr event across all divisions.

### Proximity to waterways does not fully explain flood inequality

**Fig. 4:**
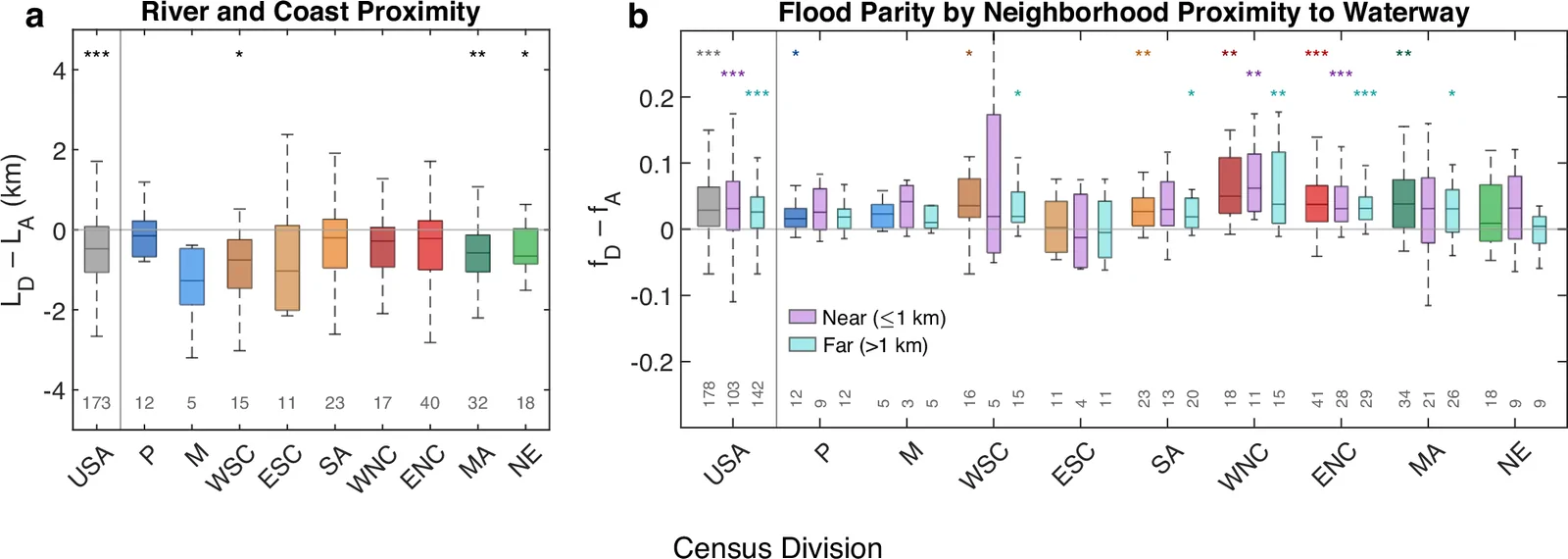
Waterway proximity and flood parity dynamics across U.S. Census divisions. **a** L_D_ and L_A_ represent the mean distance (in km) to the nearest river or coast of D- and A-graded neighborhoods in a city, respectively. Cities near a coast or river (stream order four or larger) were considered (*n* = 173). **b** Flood parity (f_D_ - f_A_) overall and partitioned by waterway proximity. f_D_ and f_A_ represent the fraction of the flooded population to total population for D- and A-graded neighborhoods in a city, respectively. Parity is shown for all neighborhoods in eligible cities (*n* = 178), as well as for subsets that consider only neighborhoods near to ( ≤ 1 km) and far from ( > 1 km) a waterway. In all cases, fractions are shown for the 100-yr flood. The boxplots show the median, interquartile range, and whiskers. Sample sizes for each group are indicated at the bottom of the axes. Outliers are not shown for visual clarity. Asterisks denote statistical significance from two-sided Wilcoxon signed-rank tests with Benjamini–Hochberg p-value adjustments (* *p* < 0.05, ** *p* < 0.005, *** *p* < 0.0005). Division codes: Pacific (P), Mountain (M), West South Central (WSC), East South Central (ESC), South Atlantic (SA), West North Central (WNC), East North Central (ENC), Middle Atlantic (MA), and New England (NE). This figure was created with MATLAB.

To explain the inequitable relations observed, we explored a foundational flooding risk factor: proximity to large waterways (Strahler order ≥ 4). While we found that D-graded neighborhoods are generally closer to large waterways (*p* < 0.0005 and Fig. 4a), this proximity is not a strong driver of flood inequality. Specifically, we observed no significant correlation between a city’s proximity disparity (L_D_ - L_A_) and its flood parity (f_D_ - f_A_) (Fig. S3). Further reinforcing this, flood inequality persisted whether neighborhoods were close to (≤1 km) or far from (>1 km) large rivers and coasts (Fig. 4b). Such consistency suggests that proximity to flooding sources alone cannot explain the observed flood inequality. Because the Fathom-US model simulates both fluvial and pluvial flooding, the persistence of disparities far from waterways implies that D-graded neighborhoods are vulnerable not only to major river and coastal flooding but also to pluvial flooding driven by stormwater runoff exceeding local drainage capacity. This points to a systemic exposure that operates independently of proximity to waterways.

### Flood inequality amplifies with increasing flood magnitude

**Fig. 5:**
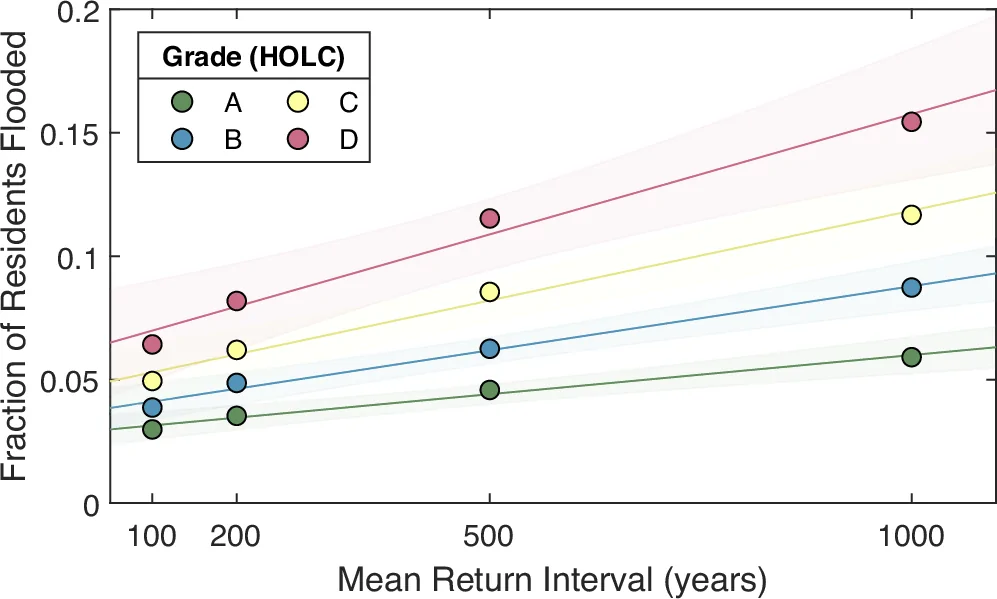
Flood exposure across mean return intervals by HOLC grade. Median flooded fractions for all 202 analyzed cities are plotted against four mean return intervals (100, 200, 500, and 1000 years) across HOLC grades (A–D). Solid lines indicate linear regressions, and shaded regions represent the corresponding 95% confidence intervals. This figure was created with MATLAB.

Flood magnitude, in this context, represents the scale of inundation for a given flood event. Higher MRIs are considered more intense and are used in this study to better understand how increasing storm intensity under climate change could affect flood exposure disparities. Our results show that the disparity in flooding grows with event magnitude (Fig. 5 and S4). Starting from a 100-yr baseline exposure of 6.4% for D-graded and 3.0% for A-graded neighborhoods, the impacts of more extreme events diverge significantly, as indicated by diverging slopes between the two grades (F = 22.73, *p* = 0.0003). As the hazard scales from a 100-yr to a 1000-yr event, A-graded neighborhoods see an additional 2.9% [1.8%, 3.9%] of residents exposed, whereas the increase in D-graded neighborhoods is roughly three times larger at 8.8% [5.1%, 12.4%].

## Discussion

This study tested the null hypothesis of no correlation between HOLC-redlining and present-day flood exposure, addressing whether HOLC grade is significantly associated with city-scale flooding and how this exposure varies with flood severity. Our analysis of digitized HOLC maps, dasymetric population distributions, and flood simulations led us to reject this hypothesis. We found that HOLC grade is a statistically significant factor in flood exposure for most cities studied. D-graded neighborhoods bear a disproportionate share of the flood burden. This research contributes to the extensive literature demonstrating HOLC-redlining’s lasting detrimental effects, which also includes disparities in urban heat^36^, food access^33^, climate risk^18^, air quality^28^, and economic mobility^32^. Whether the HOLC grading system actively caused modern divestment or merely documented pre-existing spatial inequalities^30^, our results indicate that the spatial organization of race and class codified by these maps remains linked to contemporary, unequal flood exposure. Notably, this contemporary inequity is not static. Because we found that the exposure gap amplifies with flood severity, these disparities are anticipated to worsen as climate change drives extreme precipitation, particularly in regions like the Mid-Atlantic and Midwest^37^.

The scale of present-day flood inequality is considerable: D-graded residents face worse flood exposure in a 100-yr event than A-graded residents face in a 1000-yr event, and this disparity is significant across most Census divisions (Fig. 3) and amplifies with flood magnitude (Fig. 5). The overrepresentation of D-graded neighborhoods in flood-prone areas reflects multiple interacting factors. Our proximity analysis confirms that D-graded neighborhoods are, on average, closer to large waterways than A-graded neighborhoods (Fig. 4a). However, the persistence of statistically significant flood inequality among neighborhoods situated more than 1 km from a waterway (Fig. 4b) demonstrates that geographic proximity alone is insufficient to explain the disparity. Because the disparity exists whether neighborhoods are close to a major waterway or not, D-graded neighborhoods likely experience exacerbated fluvial and pluvial flooding. This finding implicates infrastructure-related factors—such as inadequate stormwater drainage, lower investment in levee protection, and greater impervious surface cover without compensating green infrastructure—as compounding drivers of pluvial flood exposure in D-graded neighborhoods^18^. These localized pluvial risks are particularly sensitive to projected urbanization. With 89% of North Americans expected to live in cities by 2050^38^, the continued concentration of people and impervious surfaces will likely aggravate flash flood conditions and increase flood risk^39^.

Looking at the geographic component of flooding, history may provide important context that can explain why D-graded neighborhoods are more often situated closer to floodplains. During the early-to-mid 20th century, industrial development, particularly in regions like the Rust Belt—denoted in Fig. 1 by the East North Central and Middle Atlantic Census divisions—was concentrated along rivers essential for power and transport^40–42^. The influx of an industrial workforce—primarily European immigrants and Black Southern migrants—to housing near these centers resulted in dense urbanization^41^. Concurrently, increasing industrial pollution, coupled with municipal divestment in services, drove the selective suburbanization of white middle-class residents^43^. HOLC grading, heavily influenced by ethnicity, class, and employment^32^, frequently redlined these urban neighborhoods. The confluence of industrial geography, labor migration, and discriminatory housing policies placed many D-graded neighborhoods at the intersection of economic marginalization, restricted socio-economic mobility, and heightened climate hazard exposure^18,28,29,36^. This risk-compounding pattern persists: low-income housing development continues to be steered toward lower-valued, flood-prone land, and population growth within flood-prone areas has outpaced growth outside them over the last several decades^44,45^. Where these trends overlap with historically redlined neighborhoods, the legacy of HOLC grading compounds with ongoing development pressures to concentrate flood risk among the most socially vulnerable populations.

While overall flood susceptibility across the U.S. has decreased, progress remains inequitable^24^. For example, current flood management often prioritizes mitigating the potential economic impact of levee breaches, which helps to protect property but not necessarily people^46^. Historically divested areas with a lower tax base thus possess fewer resources for structural resilience retrofits and large-scale flood defense projects^18^. Higher-income municipalities or neighborhoods often possess a stronger tax base to finance flood prevention infrastructure and private insurance, enabling the existence of highly protected, resilient communities along coastlines and waterways that would otherwise present a hazard to residents^47^. This reinforces a divide wherein affluent communities become ‘risk-averse’ due to superior flood defenses and institutional memory of past floods, while economically marginalized communities are left to remain ‘risk enduring’^20^.

Our findings, like other hydrologic model-based studies^48^, have limitations. First, we analyzed flood extent, not depth, focusing on population impact rather than property damage severity. While estimating severity is important, the depth-to-damage relationships that have been established exhibit considerable spatial variation, and there is presently a lack of high-resolution data to accurately resolve the depth, population, and redlining dynamics^49^. Further research at a localized scale with high-resolution depth-to-damage data should be carried out to more deeply explore the broad trends found in this study. Second, while stormwater and flood prevention infrastructure is built into the Fathom-US model, this paper does not go further to investigate the localized role of stormwater infrastructure, largely because these data are not available at the granularity required to derive meaningful conclusions^50^. Our finding that flood inequality persists far from waterways (Fig. 4b) underscores the importance of this data gap: differences in stormwater drainage capacity, green infrastructure, and impervious surface cover are plausible drivers that cannot be tested without spatially resolved infrastructure data. Future work should address this as such data become available. Third, the dasymetric population data (based on 2016 estimates) and Fathom-US flood maps (calibrated to 2017 climate understanding) represent static snapshots, an inherent limitation of this type of analysis that may not perfectly capture present-day or future conditions. Fourth, while the Fathom-US flood model and dasymetric population model are highly accurate compared to other contemporary methods, they are both large-scale computational models whose accuracy is superseded by local surveyed flood extents or populations. As the availability of accurate topographic and hydrologic data expands, further research should be undertaken to verify the assertions made by this study.

This study advances the understanding of the link between HOLC-redlining and flood exposure by: (1) quantifying present-day flood disparity across all four HOLC grades, (2) identifying spatial variability in this vulnerability across U.S. Census divisions, (3) showing how flood severity magnifies these inequalities, and (4) demonstrating that proximity to waterways does not fully account for the observed disparities, implicating infrastructure-related factors. We underscore how policies like HOLC redlining may perpetuate environmental inequality long after their dissolution. As increasing precipitation extremes threaten to amplify existing flood inequities^37^, effective flood planning is necessary. These findings highlight the necessity for policymakers and engineers to integrate historical equity and climate projections into future infrastructural design. Our results demonstrate flood disparity between the ‘best’ and ‘hazardous’ neighborhoods of most US cities (Fig. 2); thus, adaptation plans that treat cities as spatially homogeneous will fail to achieve equitable outcomes. In the cases where equitable infrastructure is constructed, such as green infrastructure, planners would need to provide for adequate long-term maintenance to not allow inequity to redevelop over time^51^. Addressing the research gap on the equity implications of flood policy is needed^52^. Future work, spurred by our findings, should focus on filling the policy research gap and translating scientific findings into actionable steps to reduce flood disparities nationwide.

## Methods

Several datasets are necessary to analyze the relationship between historically redlined populations and their modern-day flooding exposure (Fig. 1). The core datasets used in this study are (1) HOLC-redlining maps, (2) dasymetric population data, (3) hydrodynamic flood simulations, and (4) water feature data. The intersection of these datasets is the primary interest of this study. Each core dataset is described in detail below, followed by how each was used in the analysis.

### HOLC redlining maps

The HOLCredlining data used in this study were obtained from Not Even Past: Social Vulnerability and the Legacy of Redlining, a digital database created by the University of Richmond’s Digital Scholarship Lab and the National Community Reinvestment Coalition^53^. Their work is derived from similar projects, such as Mapping Inequality: Redlining in New Deal America, also from the University of Richmond^54^. The dataset obtained from the open-source database came in the form of a shapefile for the contiguous United States. The dataset contains 202 cities that are composed of 9716 neighborhoods. The HOLC-redlining shapefile included the grade (A, B, C, or D) of each neighborhood and the written descriptions provided by assessors at the time of grade assignment. First was A, colored green on the maps, which was given to ‘best neighborhoods where access to credit was granted with little resistance'. Second was B, colored blue, which was granted to ‘still desirable’ areas. Third was C, colored yellow, which signaled that lenders ought to exercise caution before lending to residents and that the neighborhood was ‘definitely declining’. Finally, the lowest grade was D, colored red, and was given to neighborhoods considered ‘hazardous’. In addition to the categorical grades, each neighborhood assessment was accompanied by qualitative descriptions written by the assessors^55^. For our study’s purposes, only the grade was used as a categorical variable to define neighborhoods.

### Dasymetric population data

One core tenet of our study is the focus of our analysis on populated areas. To spatially determine which areas were populated and which were not, we used the updatable gridded lightweight impervious (UGLI) dasymetric population dataset^23^. UGLI is a combination of two datasets: US Census Bureau population data and National Land Cover Database (NLCD) imperviousness data. Areas that the authors considered to be unpopulated were forests, fields, pastures, roadways, commercial buildings, and industrial buildings. The remaining impervious cells that were likely associated with housing were cross-referenced against the Microsoft US Building Footprint dataset. For these populated cells, the population was estimated by apportioning the total 5-year population as generated by the 2016 American Community Survey. For forests, fields, pastures, roadways, commercial buildings, and industrial buildings, the population was set to zero. The resulting model output was a raster with population density values at a 30-m resolution. The accuracy of the UGLI was compared to predictions made by models from the US Environmental Protection Agency, Microsoft, and Facebook^23^, and the original authors demonstrated that UGLI performs favorably.

### Flood model simulations

Flood mapping was performed using the Fathom-US modeled dataset^12^, which was accessed through an academic license. Figure 1 and S1 demonstrate examples of the 10-m resolution flooding product. The Fathom-US model simulates two-dimensional hydrodynamics for all of the contiguous US and includes fluvial, pluvial, and coastal flooding in its results^12^. The model was evaluated on publicly available data over the last century up until 2020, as well as flood maps produced by the Iowa Flood Center^12^. The Fathom-US model also includes the most complete database of flood defenses^13^. The Fathom model has near-complete coverage of the contiguous US compared to just 60% coverage provided by FEMA flood maps^13^. The Fathom-US model features simulations at ten different return periods (MRIs), ranging from 1:5 year to 1:1000 year. This important distinction allows us to get a more complete map of flooding extent across many intervals, spatial locations, and scales. We analyze four MRIs that are used to describe infrequent, extreme events: 100-, 200-, 500-, and 1000-yr.

### Flowline and coastline data

To assess the proximity of HOLC grade neighborhoods to waterways, we used a spatial representation of flowlines and coastlines from the United States Geological Survey (USGS) NHDPlus High Resolution dataset^56^. For the proximity analysis, the 202-city dataset was restricted to cities that 1) include both A- and D-graded neighborhoods, and 2) were within 5 km on average of a coastline or river feature with Strahler stream order greater than or equal to 4. The first criteria reduced the city number down to 178, and the second criterion reduced it to 173.

Figure S5 shows the methodology for calculating the mean average distance (L) of A- and D-graded neighborhoods from the nearest identified water feature. For each city, and for each HOLC grade, the centroid of every individual neighborhood polygon of that grade was calculated. The distance from each neighborhood centroid to the nearest qualifying water feature was then determined. These distances were then averaged for all A-graded neighborhoods within the city to yield L_A_, and similarly for all D-graded neighborhoods to yield L_D_. To determine whether A- or D-graded neighborhoods were more proximal across all cities (i.e., L_D_ ≠ L_A_), we employed a Wilcoxon signed-rank test with Benjamini–Hochberg p-value adjustment^57^.

### Analyzing the affected population

First, the total population for each neighborhood was calculated by summing the dasymetric population raster within each neighborhood’s boundaries. The total population for a city is the sum of the population of all neighborhoods. The city populations listed are for those in the HOLC-defined neighborhoods as originally drawn, and thus population growth in the city outside of these neighborhoods is not considered, nor is any redrawing of municipal boundaries.

To calculate the flooded population for each MRI, we overlaid the populated neighborhood raster and the Fathom-US model products. For this study, we consider only the extent (presence) of flooding rather than the depth at any given point. The population that resided within the area of non-zero inundation was tabulated and reported by neighborhood. The population residing within the inundation boundary for a given MRI was considered to be the ‘flooded population’. The depth of flooding at any given point was not considered in this analysis.

For each city, we assessed whether a neighborhood grade is over- or under-represented in its share of the flood burden. The share of the flood burden was calculated by dividing the flooded population for a particular neighborhood grade by the total flooded population in a city, represented by f_flood-pop_. This fraction was compared to the neighborhood grade’s share of the overall city population, represented by f_total-pop_. Our null hypothesis assumed an equal urban flooding scenario (i.e., f_flood-pop_/f_total-pop_ = 1) where HOLC-graded neighborhoods have an equal chance (50%) of being overrepresented (f_flood-pop_/f_total-pop_ > 1) or underrepresented (f_flood-pop_/f_total-pop_ < 1) in sharing the flood burden. To test this hypothesis, we employed a binomial sign test for each of the HOLC grades with Benjamini–Hochberg p-value adjustment^57^.

We define a pairwise measure of flood parity between the A and D neighborhood grades in a city as f_D_ – f_A_. Here, f represents a ratio of the flooded population (e.g., D_flood_) to the total population (e.g., D_total_) for a neighborhood grade in a city. Our null hypothesis was that of perfect flood parity, i.e., f_D_ = f_A_. To test this hypothesis for the US as a whole and for nine US Census divisions, we employed Wilcoxon signed-rank tests with Benjamini–Hochberg *p*-value adjustments^57^.

To disentangle the influence of geographic proximity from other systemic factors, we repeated the flood parity analysis on spatial subsets, restricting the data to neighborhoods located near ( ≤ 1 km) and far ( > 1 km) from a major waterway. Furthermore, we evaluated the direct relationship between flood parity (f_D_ – f_A_) and proximity (L_D_ – L_A_) by calculating the Spearman rank correlation coefficient (*ρ*) and fitting a robust linear regression. This correlation analysis was conducted globally for the US and regionally for each Census division.

To assess how flood inequalities may change with flood magnitude, we developed linear regressions between the flooded population in each HOLC neighborhood grade versus MRI. The slope of the regressions and their 95% confidence intervals are reported. An analysis of covariance (ANCOVA) test was conducted to test significant differences in slopes between the neighborhood grades. The F-statistic and p-value are reported for the ANCOVA test. All statistical tests were done at a significance level of 0.05. All geospatial analysis was conducted with ArcMap 10.8.2, and all statistical analysis was performed with MATLAB 2024a.

### Reporting summary

Further information on research design is available in the Nature Portfolio Reporting Summary linked to this article.

## Supplementary information

Supplementary Information

Reporting Summary

Transparent Peer Review file

## Data Availability

Flood modeling products were retrieved through an academic license from Fathom-US at https://www.fathom.global/. Dasymetric population data were downloaded from the Updatable Gridded Lightweight Impervious dataset at 10.5281/zenodo.5750665. HOLC-redlining maps were retrieved from the Not Even Past dataset at https://dsl.richmond.edu/panorama/redlining/data. The aggregated neighborhood- and city-scale data that support the findings of this study are available online at the following Open Science Framework repository link: 10.17605/OSF.IO/JD3E5.
