# Supplementary material for: Disproportionate exposure to extreme floods in historically redlined U.S. communities
Source: Nat Commun. 2026 Jul 23;17:8990. doi: 10.1038/s41467-026-76024-2 (PMC13507297; doi:10.1038/s41467-026-76024-2)
Supplement: Supplementary file 1 [file 41467_2026_76024_MOESM1_ESM.pdf]

SUPPLEMENTAL MATERIAL

Disproportionate exposure to extreme floods in historically redlined U.S. communities

Elizabeth Appel[1,2], S. Amin Enderami[1], Elaina J. Sutley[1], and Admin Husic[1,3*]
[1]Department of Civil, Environmental and Architectural Engineering, University of Kansas, Lawrence, KS, USA
[2]Department of Engineering, University of Cambridge, Cambridgeshire, UK
[3]Department of Civil and Environmental Engineering, Virginia Tech, Blacksburg, VA, USA

*Corresponding author: husic@vt.edu

Supporting information contains 6 pages (S1 through S6): this cover and five figures.

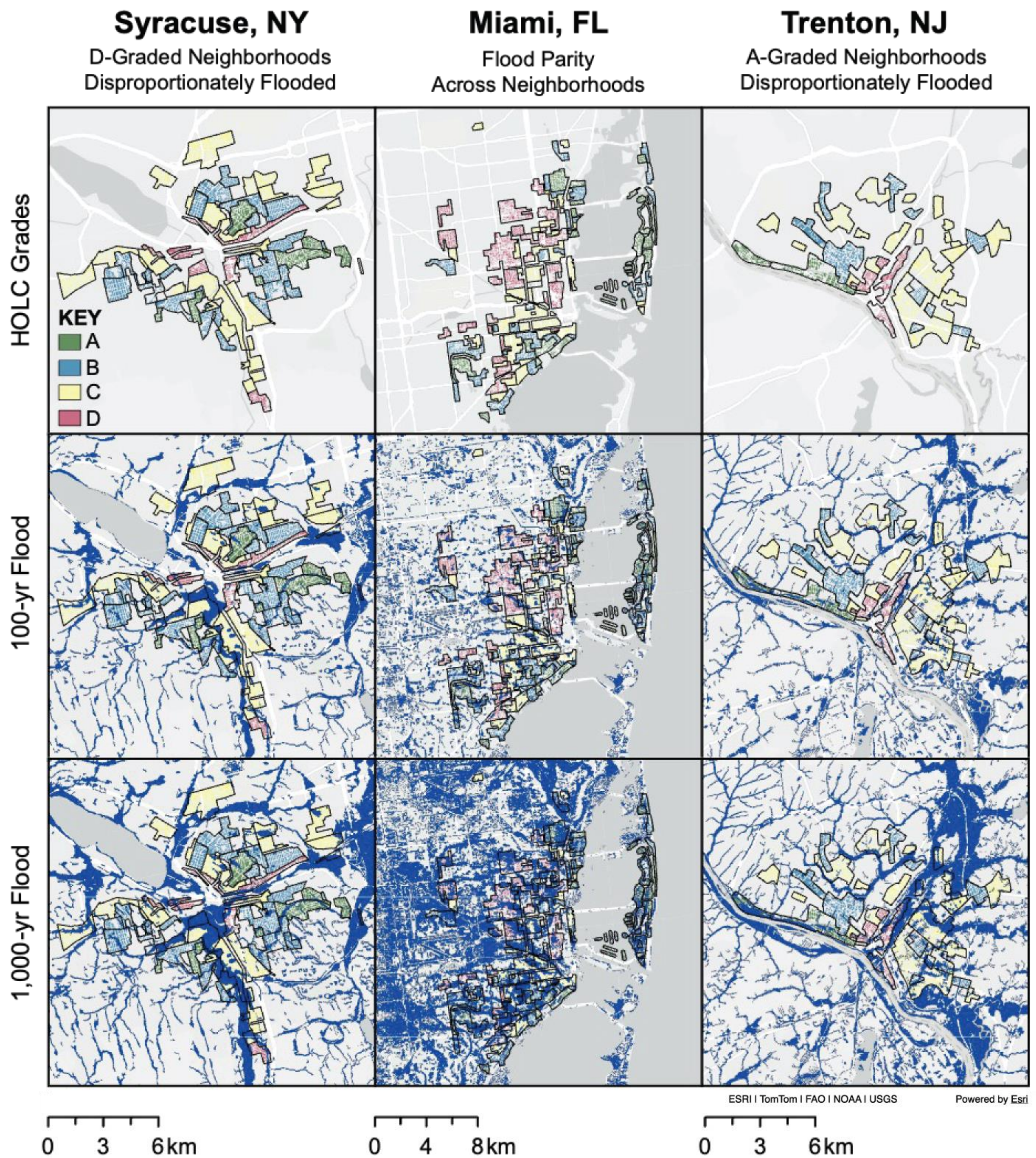

Figure S1: Flood inundation distributions for Syracuse (NY), Miami (FL), and Trenton (NJ) for 100-yr and 1,000-yr return intervals. Syracuse is characterized by disproportionate flooding of D-Graded (redlined) communities, whereas Trenton shows disproportionate flooding of A-Graded communities. Miami has relative parity in flood impacts. Black lines outline the neighborhoods as defined by the HOLC. Color-coded cells within neighborhoods represent dasymetrically populated cells. The basemaps used in this figure are the intellectual property of Esri and are used herein under license. Copyright © 2025 Esri and its licensors. All rights reserved.

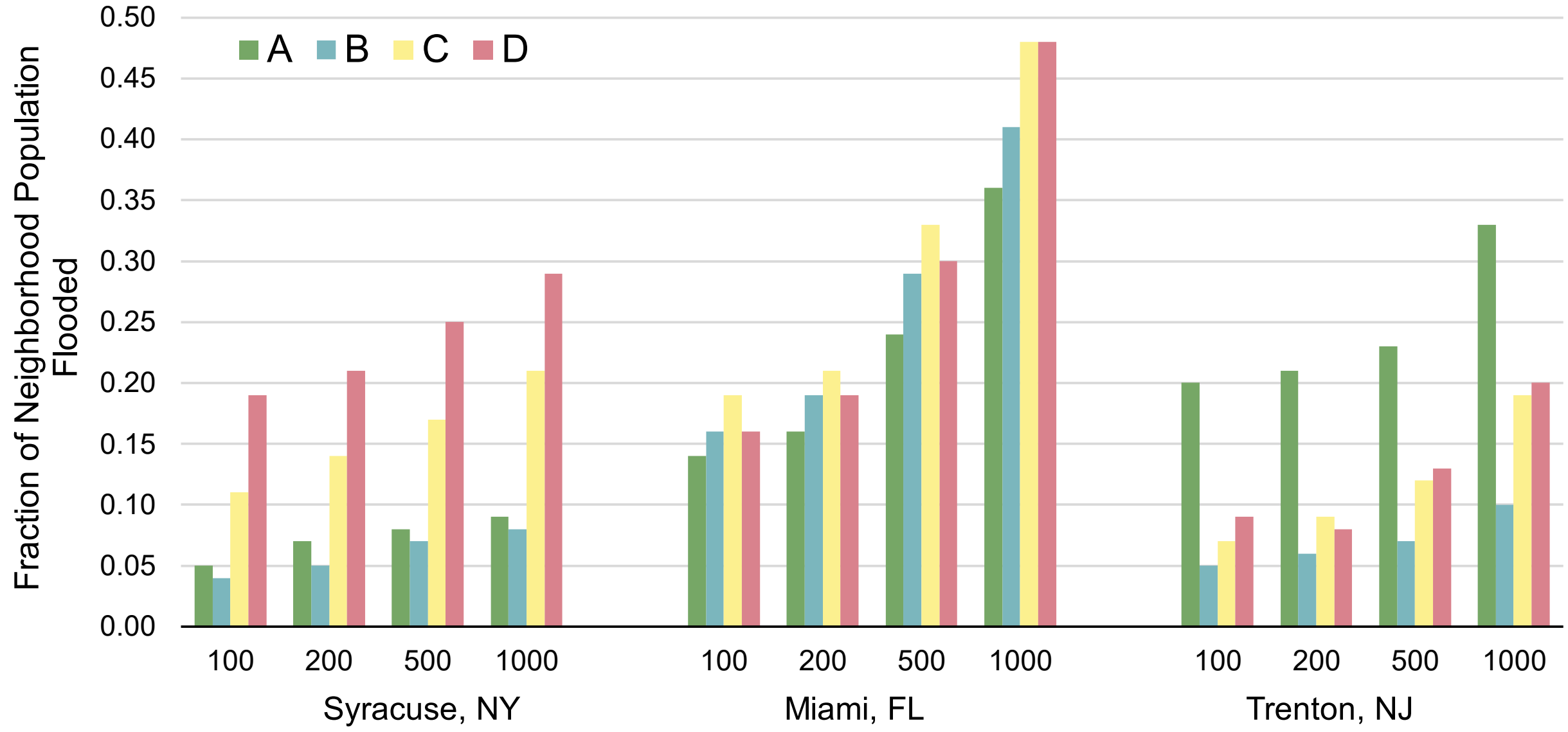

Figure S2: Flood inundation results for Syracuse (NY), Miami (FL), and Trenton (NJ). The x-axis refers to the mean return interval (MRI) of a flood event. The flooded fraction represents the fraction of residents living in the graded neighborhood who are impacted by flooding. Miami has relative parity in flood impacts. Syracuse is characterized by disproportionate flooding of D-Graded communities, whereas Trenton shows disproportionate flooding of A-Graded communities.

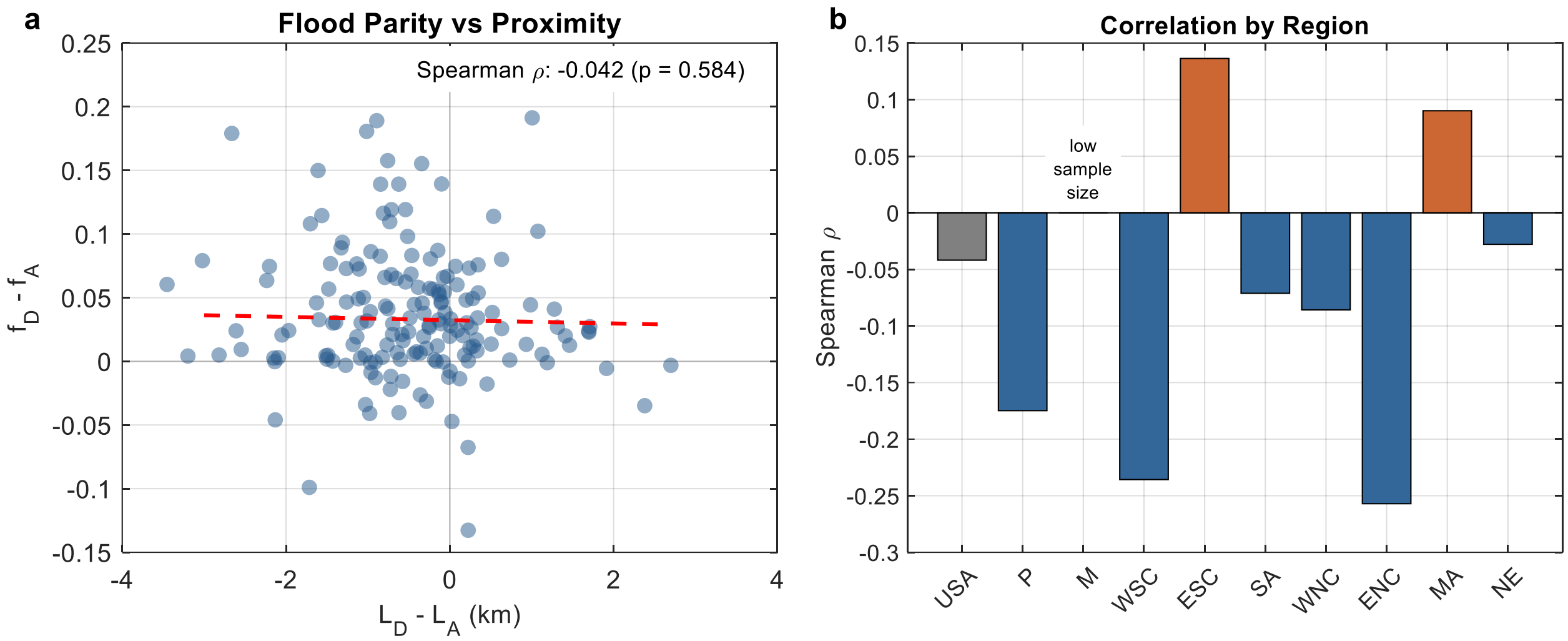

Figure S3. Correlation between flood parity and proximity disparity for the USA and Census divisions. f_D and f_A represent the fraction of the flooded population to total population for D- and A-graded neighborhoods in a city, respectively. Fractions are shown for the 100-yr flood. L_D and L_A represent the mean distance (in km) to the nearest river or coast of D- and A-graded neighborhoods in a city, respectively. Only cities meeting the criteria for both parity and proximity analyses were considered. (a) Scatter plot showing the relationship between proximity disparity ($L_D - L_A$) and flood parity ($f_D - f_A$) across all eligible cities. The red dashed line represents a robust linear fit, with the overall Spearman rank correlation coefficient (ρ) and p-value annotated. (b) Spearman rank correlation coefficients (ρ) calculated for the USA and individually for each Census division. Bar colors denote positive (orange) and negative (blue) correlations. Divisions with a sample size of $n \leq 5$ were excluded from the regional analysis due to insufficient data. Division codes: Pacific (P), Mountain (M), West South Central (WSC), East South Central (ESC), South Atlantic (SA), West North Central (WNC), East North Central (ENC), Middle Atlantic (MA), and New England (NE). This figure was created with MATLAB (https://www.mathworks.com/products/matlab.html).

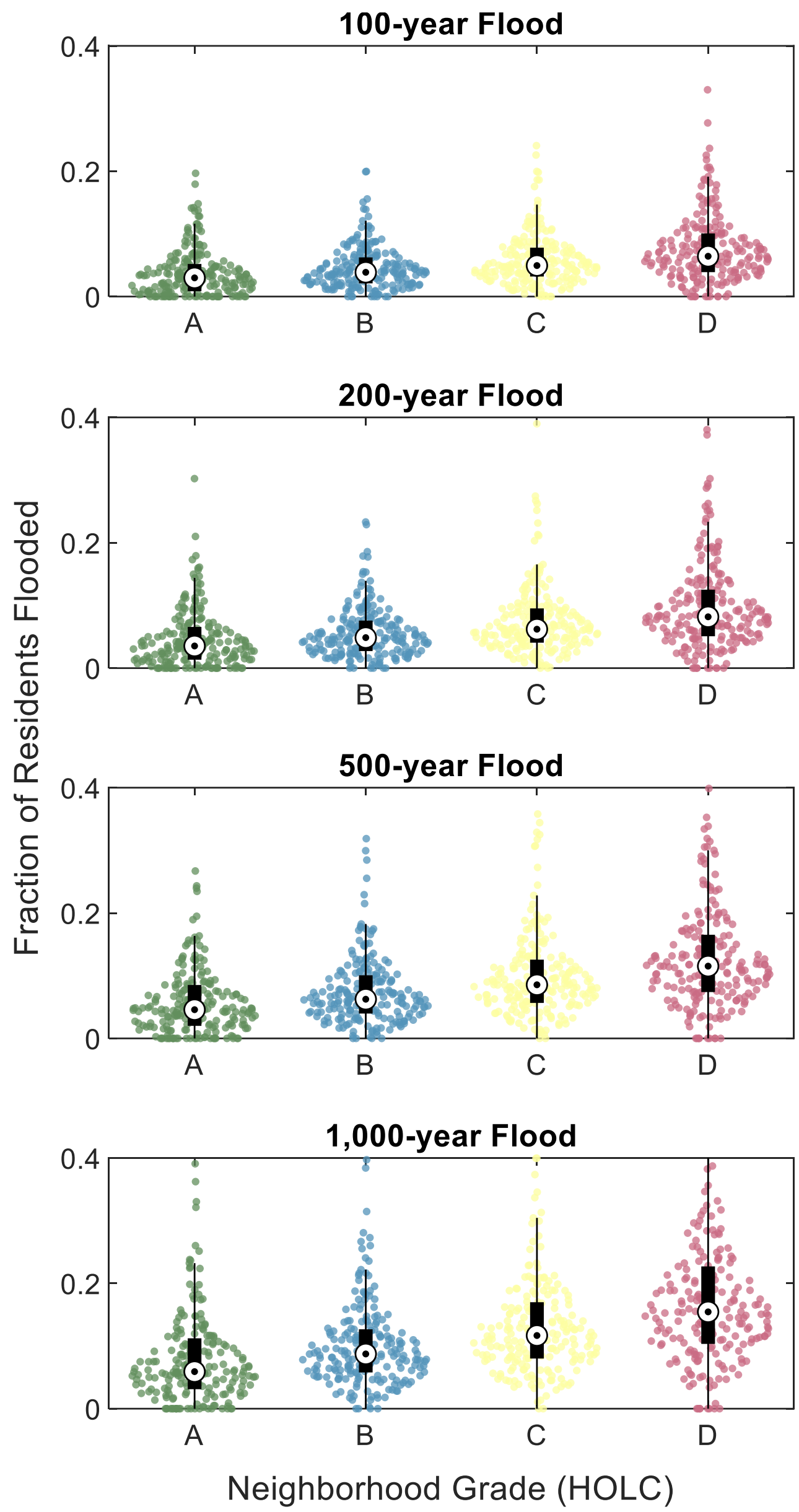

Figure S4: Swarm chart and boxplot of the fraction of residents within each neighborhood grade that experience some amount of flooding for 100-, 200-, 500-, and 1,000-year mean return interval floods. Each dot in the swarm chart represents all neighborhoods of a certain grade for each of the 202 communities in our analysis. The boxplot indicates a median (circle), the inter-quartile range (bars), and whiskers (lines). This figure was created with MATLAB (https://www.mathworks.com/products/matlab.html).

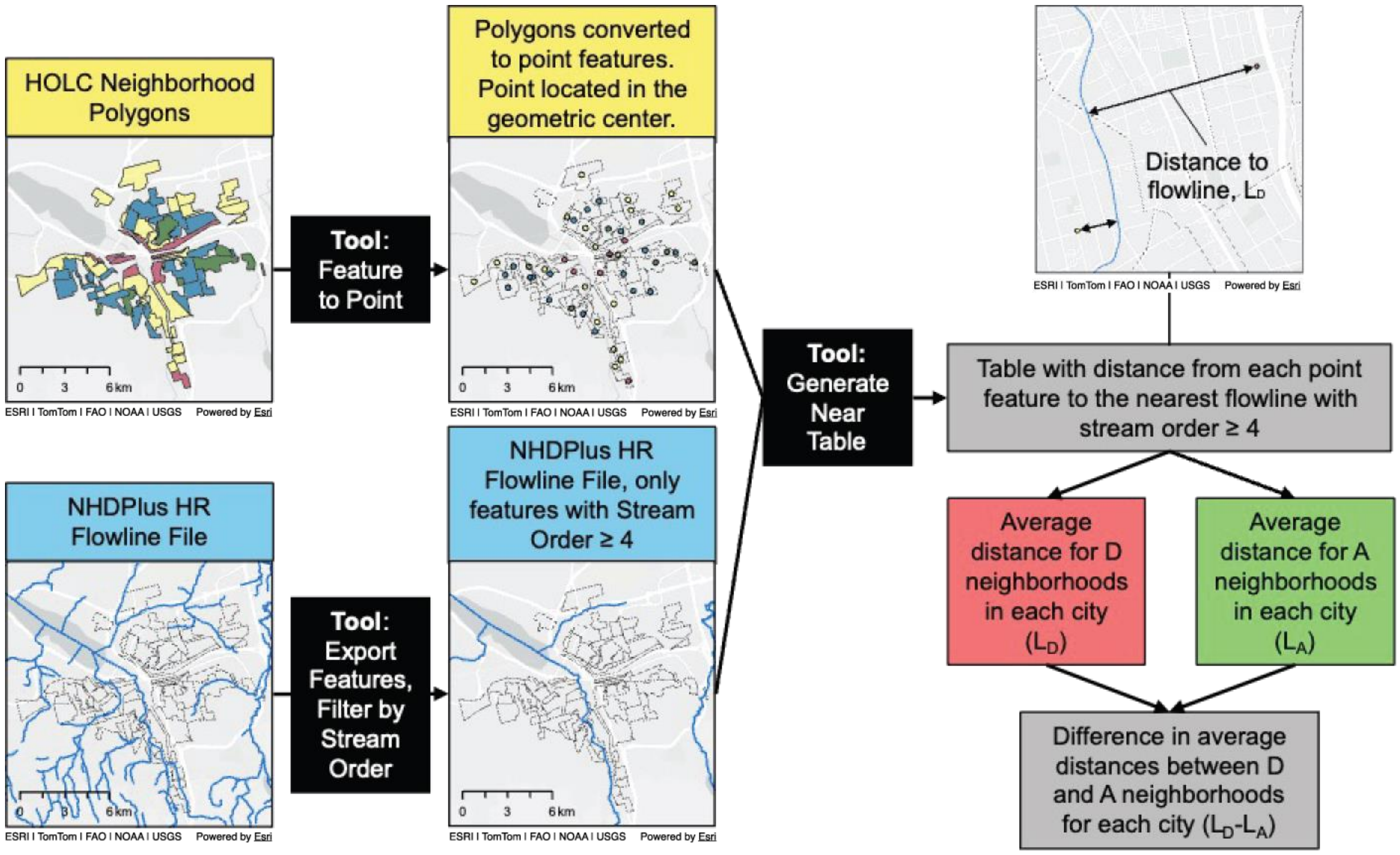

Figure S5: Process diagram for calculation of neighborhood proximity to waterway with stream order greater than or equal to 4. The streamline file comes from the United States Geological Survey NHDPlus High-Resolution database. The basemaps used in panels (b) and (c) are the intellectual property of Esri and are used herein under license. Copyright © 2025 Esri and its licensors. All rights reserved.
