# Supplementary material for: Disproportionate exposure to extreme floods in historically redlined U.S. communities
Source: Nat Commun. 2026 Jul 23;17:8990. doi: 10.1038/s41467-026-76024-2 (PMC13507297; doi:10.1038/s41467-026-76024-2)
Supplement: Supplementary file 2 [file 41467_2026_76024_MOESM2_ESM.pdf]

nature portfolio

Corresponding author(s): Admin Husic

Last updated by author(s): Jun 3, 2026

Reporting Summary

Nature Portfolio wishes to improve the reproducibility of the work that we publish. This form provides structure for consistency and transparency in reporting. For further information on Nature Portfolio policies, see our Editorial Policies and the Editorial Policy Checklist.

Statistics

For all statistical analyses, confirm that the following items are present in the figure legend, table legend, main text, or Methods section.

n/a	Confirmed	
☐	☒	The exact sample size (*n*) for each experimental group/condition, given as a discrete number and unit of measurement
☒	☐	A statement on whether measurements were taken from distinct samples or whether the same sample was measured repeatedly
☐	☒	The statistical test(s) used AND whether they are one- or two-sided<br>*Only common tests should be described solely by name; describe more complex techniques in the Methods section.*
☐	☒	A description of all covariates tested
☐	☒	A description of any assumptions or corrections, such as tests of normality and adjustment for multiple comparisons
☐	☒	A full description of the statistical parameters including central tendency (e.g. means) or other basic estimates (e.g. regression coefficient) AND variation (e.g. standard deviation) or associated estimates of uncertainty (e.g. confidence intervals)
☐	☒	For null hypothesis testing, the test statistic (e.g. *F*, *t*, *r*) with confidence intervals, effect sizes, degrees of freedom and *P* value noted<br>*Give P values as exact values whenever suitable.*
☒	☐	For Bayesian analysis, information on the choice of priors and Markov chain Monte Carlo settings
☒	☐	For hierarchical and complex designs, identification of the appropriate level for tests and full reporting of outcomes
☒	☐	Estimates of effect sizes (e.g. Cohen's *d*, Pearson's *r*), indicating how they were calculated

Our web collection on statistics for biologists contains articles on many of the points above.

Software and code

Policy information about availability of computer code

Data collection	All data used for this project were obtained from existing sources, thus no data collection was performed directly by the authors of this study. The sources of the data are identified in the Methods section and Data Availability of the manuscript.
Data analysis	Geospatial analysis was conducted in ArcMap 10.8.2. Statistical analysis was performed in MATLAB 2024a using the Statistics and Machine Learning Toolbox. Figures were generated in ArcMap 10.8.2.and MATLAB 2024a.

For manuscripts utilizing custom algorithms or software that are central to the research but not yet described in published literature, software must be made available to editors and reviewers. We strongly encourage code deposition in a community repository (e.g. GitHub). See the Nature Portfolio guidelines for submitting code & software for further information.

Data

Policy information about availability of data

All manuscripts must include a data availability statement. This statement should provide the following information, where applicable:

- Accession codes, unique identifiers, or web links for publicly available datasets
- A description of any restrictions on data availability
- For clinical datasets or third party data, please ensure that the statement adheres to our policy

The redlining data used in this study was obtained from Not Even Past: Social Vulnerability and the Legacy of Redlining, a digital database created by the University

of Richmond's Digital Scholarship Lab and the National Community Reinvestment Corporation (https://dsl.richmond.edu/socialvulnerability/). Flood mapping was performed using the Fathom-US dataset, which was accessed through an academic license (https://www.fathom.global/product/global-flood-map/us-flood-map/).

Human research participants

Policy information about studies involving human research participants and Sex and Gender in Research.

Reporting on sex and gender	N/A
Population characteristics	N/A
Recruitment	N/A
Ethics oversight	N/A

Note that full information on the approval of the study protocol must also be provided in the manuscript.

Field-specific reporting

Please select the one below that is the best fit for your research. If you are not sure, read the appropriate sections before making your selection.

☐ Life sciences ☐ Behavioural & social sciences ☒ Ecological, evolutionary & environmental sciences

For a reference copy of the document with all sections, see nature.com/documents/nr-reporting-summary-flat.pdf

Ecological, evolutionary & environmental sciences study design

All studies must disclose on these points even when the disclosure is negative.

Study description	We investigated how modern-day flooding is distributed across redlined and non-redlined neighborhoods.
Research sample	Starting in the 1930s, the Home Owners Loan Corporation (HOLC) began a process termed redlining where they determined the suitability of certain neighborhoods for mortgage loans. Digitized maps of these neighborhoods exist for 202 cities. We analyze the flood impacts for all 202 cities in the digitized database.
Sampling strategy	All cities and their respective neighborhoods in the digitized database.
Data collection	All data used for this project were obtained from existing sources, thus no data collection was performed directly by the authors of this study.
Timing and spatial scale	All data used for this project were obtained from existing sources, thus no data collection was performed directly by the authors of this study.
Data exclusions	No cities that are in the digitized database were excluded from analysis.
Reproducibility	N/A
Randomization	N/A
Blinding	N/A

Did the study involve field work? ☐ Yes ☒ No

Reporting for specific materials, systems and methods

We require information from authors about some types of materials, experimental systems and methods used in many studies. Here, indicate whether each material, system or method listed is relevant to your study. If you are not sure if a list item applies to your research, read the appropriate section before selecting a response.

Materials & experimental systems

n/a	Involved in the study
☒	☐ Antibodies
☒	☐ Eukaryotic cell lines
☒	☐ Palaeontology and archaeology
☒	☐ Animals and other organisms
☒	☐ Clinical data
☒	☐ Dual use research of concern

Methods

n/a	Involved in the study
☒	☐ ChIP-seq
☒	☐ Flow cytometry
☒	☐ MRI-based neuroimaging
