# Supplementary material for: Disproportionate exposure to extreme floods in historically redlined U.S. communities
Source: Nat Commun. 2026 Jul 23;17:8990. doi: 10.1038/s41467-026-76024-2 (PMC13507297; doi:10.1038/s41467-026-76024-2)
Supplement: Supplementary file 3 [file 41467_2026_76024_MOESM3_ESM.pdf]

nature portfolio

Peer Review File

Disproportionate exposure to extreme floods in historically redlined U.S. communities

Corresponding Author: Dr Admin Husic

This manuscript has been previously reviewed at another journal. This document only contains information relating to versions considered at Nature Communications. Mentions of the other journal have been redacted.

This file contains all reviewer reports in order by version, followed by all author rebuttals in order by version.

Version 0:

Reviewer comments:

Reviewer #1

(Remarks to the Author)
This manuscript presents an interesting study that connects historical public policy with current flood risk exposure. The authors analyze neighborhoods in 202 U.S. cities that were historically graded by the Home Owners' Loan Corporation and demonstrate that residents in formerly redlined (D-graded) areas experience disproportionately higher flood exposure today.

The topic of this research is highly relevant as it links historical urban development, social inequality, and environmental vulnerability. The study offers new insights for equitable flood management and urban planning by revealing the persistent impacts of discriminatory housing policies on present-day climate risks.

The analysis framework is clear and straightforward. The data sources, modeling approach, and results are easy to follow, and the conclusions are well supported by the evidence. The manuscript is well written, and it tells a compelling story without relying on complex technical methods, which makes it accessible to a broad readership.

Overall, we did not identify major flaws in the design or interpretation, and recommend the manuscript for publication in Nature Communications after the following comments are addressed.

Major comments

The study uses the Fathom-US flood model, which provides high-resolution coverage of fluvial (river-driven), pluvial (rainfall-driven), and coastal flooding across the U.S. for multiple return intervals. The results in Figure 2 clearly demonstrate that the proximity of communities to waterways plays a key role in determining flood exposure. D-graded neighborhoods experience a greater share of the flood burden in 146 cities, largely due to their closer proximity to rivers or coasts. However, it appears that pluvial flooding—which results from rainfall-generated overland flow and ponding before runoff enters watercourses or drainage systems—has not been fully represented in the analysis or discussion. Because pluvial flooding is not necessarily correlated with distance to waterways, proximity alone may not adequately capture its influence on neighborhood-level exposure. It would be valuable to clarify whether pluvial flooding contributes differently to exposure patterns across neighborhood grades. For instance, do the results suggest that pluvial flooding affects all communities more evenly, or could it also reinforce disparities in certain urban contexts?

Flood depth also plays an important role in determining flood damage and exposure severity. It is not clear to us how the flood extents were defined in the Fathom-US simulations used in this study. I assume a certain threshold of inundation depth (0, 0.05m or 0.1m?) was applied to classify areas as flooded. Clarifying this criterion would help readers better understand how exposure was quantified. Moreover, in cities following the Syracuse pattern, the manuscript notes that communities located near river pathways are more susceptible to flooding. If inundation depth—rather than extent alone—were integrated into the analysis, the disparity in exposure between A- and D-graded neighborhoods might become even more pronounced. Clarifying this could provide additional insight into flood risk inequality.

Minor comments

Line 18: What is meant by ‘gap in experienced flooding’? Up to now, the abstract talks about synthetic floods (e.g. 1000-year flood) and not real floods.

Similarly: Lines 201, 219, 288: “… experienced flooding …”: Why experienced? To use this term, we think you should talk about actual, observed events that people have experienced. 100- and 1000-year floods are synthetic entities and not real floods.

Lines 140-142 and Figure 2: There is a negative correlation between flood parity and river/coast proximity (in Figure 2). Have you tested the significance of this statistical relationship (fD-fA and LD-LA) directly? Would it could be interesting to plot these two variables against each other – hoping the relationship could be interpreted?

Line 204: We are unsure about the statement “… for every 1,000-year increase in MRI, an additional 3.2% [2.0%, 4.3%] of residents are exposed …”. Do you mean: … for an increase from 100- to 10000-year flooding, an additional 3.2% …?

Line 237: what exactly is meant by “… navigating resources …”?

Line 242: The statement that D-graded neighborhoods are without adequate flood infrastructure: Is this statement supported by data or rather a (highly plausible) hypothesis? Please clarify.

Line 280: Please explain the difference between the terms “equality” and “equity”. Many readers might not be aware of it.

Figure S2: The title for the x-axis is missing (We assume it should be mean return interval (MRI) of flooding).

Reviewer #2

(Remarks to the Author)
I co-reviewed this manuscript with one of the reviewers who provided the listed reports. This is part of the Nature Communications initiative to facilitate training in peer review and to provide appropriate recognition for Early Career Researchers who co-review manuscripts.

Reviewer #3

(Remarks to the Author)
This version has undergone major revisions from several reviewers, which clearly required time and effort! I have a couple of comments here that might help finalize the manuscript:
- The abstract describes HOLC incorrectly... it was not assessing for lending as either A OR D. There is an assumption the reader is already familiar with HOLC, otherwise, this is misleading!
- Maybe something to concern for the abstract, but line 77 has, what I think, is a critical sentence that better sets the stage for this work: "quantify the scale of flood inequity as borne by people, rather, than property..."
- The qualifier "experienced" is used a lot to describe flooding, including the abstract... is there an un-experienced flooding? Can you eliminate to be more concise / less wording and still retain the importance of the statement?
- I have trouble with the focus on A and D only. There is research that shows that, in coastal cities, B are better off than A because A communities are directly on the coast and their affluency affords them the ability to manage flood risk better than C and D. Alternatively, C communities can be as bad as D, if not worse (e.g., Napieralski et al., 2024). I assume the reason for A and D is to compare the two "furthest from each other" grades, but ignoring B and C may hide some relevant patterns. You also have figures that show A, B, C, and D.
- This work is solid, and adds to a growing body of work on flood inequities in a changing environment. I would say this manuscript reveals some important spatial trends and projections, but flood inequity is already well known. This includes impact of impending changes in climate, and many of those papers are not cited (e.g. Mason et al., 2017, Rickless et al., 2020, Shepherd and KC, 2015, Wing et al., 2022), nor is there a sufficient introduction to this issue (part of the "widening" of flood exposure).
- I think the citation for economic losses is outdated, as there is more up to date numbers than those from 2015! Please update with most recent estimates to be relevant.
- "Flooding is driven by a combination of geologic, climatic, and hydrologic factors"... ok, but what about development, especially on floodplains or loss of floodplains, and stormwater infrastructure limitations / failures? There is a conversation in that paragraph about land use change, which is not really one of those three factors, as it is mostly controlled by human behavior. Please be clearer in statements, as the readership for this journal is quite diverse.
- I would refer to Syracuse as a "case study" to prove or illustrate the concept for all 202 cities. Is that what role it plays in this manuscript? I am not sure why Syracuse was selected, especially since it can appear biased or narrow focused, as cities along a coast might show different patterns (sometimes A communities are positioned with the best views, adjacent coastlines, whereas A and B in inland cities, like Syracuse, are more distal from rivers). I just don't get the jump to Syracuse, especially since it is not mentioned previously and takes up some space that may limit depth or background elsewhere in the manuscript.
- Line 100: "relatively less flooding"... means what? If you use Syracuse, can you give the reader some idea as to what this means (quantify it)?
- Phrases like "As will be shown in the upcoming analyses" (line 114) is wordy - if eliminated, the sentence still makes sense.
- Figure 1, showing the regions or census divisions, is an improvement from earlier version - it helps the reader understand

Figure 2 better! Nice!
- Not Even Past is mentioned a lot, but no detail is given (just mentioned in line 67-68 as a source of digitized maps). The citation is odd, maybe it is correct, but I have seen different versions for the redlining data.
Line 140 - still discussing Syracuse as an example, as if they are the only one that is necessary to show these relationships / patterns. Again, is this true for coasts, as flooding is perhaps driven by a combination of fluvial and coastal, depending on location?
- Line 161 mentions C-graded communities. Why?
- I think the flood inequality grows with flood intensity section is important! There is a generalization of projected rainfall patterns, implying all areas will experience more intense storms. This is true for northern states and east coast states... the others may see less precipitation but the occasional "weather whiplash" that brings the occasional heavy rain event, perhaps driven by ENSO.
- Line 221-222 is awkwardly written. There are a number of sentences like this that can be more direct, clear, and concise.
- Line 254: "situated"? Maybe "placed"?
- I've read through a number of articles in this journal to see writing style, and very few (if any), relied so heavily on "we" and "us". I prefer scientists limit use of first person writing when describing their work and there is too much first person writing in this manuscript.
- Line 369: What is CONUS? Spell out acronym first time it is used!
- Line 389: Why use 2016 ACS data? Nothing more recent?

Reviewer #4

(Remarks to the Author)
The paper examines differences in modern-day flood exposure across neighborhoods historically graded by the Home Owners' Loan Corporation (HOLC) in 202 U.S. cities. The authors show that neighborhoods historically designated as hazardous (D-graded) experience significantly higher flood exposure today than neighborhoods historically designated as desirable (A-graded), and that this disparity widens as floods become more severe.

Overall, the authors have satisfactorily addressed the reviewers' concerns. I have two remaining points that should be addressed before publication.

The authors acknowledge that neighborhoods graded as hazardous were historically located closer to rivers, coasts, and other flood-prone areas, and the newly added proximity analysis reinforces this point. As a result, the observed disparities in flood exposure may reflect a persistent geographic disadvantage that was later codified by HOLC grading, rather than an effect of redlining itself. I recommend making this limitation more explicit in the Discussion by clarifying that the analysis does not distinguish between flood disparities driven by pre-existing geography and those driven by redlining as a policy intervention per se.

Besides acknowledging this, the paper would benefit from at least providing some exercises trying to disentangle the causal role of HOLC from that of pre-existing geographic differences. One way to do this would be to compute the gap f_D-f_A for a sample of neighborhoods matched on geographic characteristics. By construction, this would isolate differences associated with HOLC grading from those driven by underlying geography. It would be helpful to see how the baseline estimates of disparities in f_D-f_A vary when the analysis is restricted to this more comparable sample.

Another way of saying this is that the paper tests the null that HOLC-graded areas are not differentially exposed to flooding. This null is easily rejected, which is not surprising given that HOLC areas differ systematically in geography. A more informative null would ask whether areas graded by HOLC with characteristics similar to those of non-HOLC areas are not differentially exposed to flooding. This gets to the heart of the distinction between geography and the direct effects of redlining.

Version 1:

Reviewer comments:

Reviewer #1

(Remarks to the Author)
The authors have responded well to all reviewer comments. I feel that the revision strengthened the already strong manuscript. I recommend publication.

I have only 2 very minor issues:

(1) Line 40 and 404: Pluvial flooding and flash flooding are set equal, but there is a slight difference between these 2 types of flooding. Typical definitions are:
- Pluvial flooding refers to flooding caused by extreme rainfall overwhelming drainage systems or ground absorption, independent of rivers, often called surface water or rainfall flooding.
- Flash floods are sudden high-velocity floods from intense rain (or other triggers like dam breaks), occurring within minutes to hours (typically < 6h).
I propose that the authors only use the term pluvial flooding and delete the term flash flooding (also in line 257).

(2) Use of equity and equality: In my earlier review, I have asked to explain the difference between the terms “equality” and “equity”. Often, there is a difference between these 2 terms, but probably not for everybody. The authors responded “… This line has been removed altogether as it ventured into an area outside of our core findings…” While this line has been removed, the general issue still persists. The manuscript contains 21 times the term equality (or inequality) and 9 times the term equity (or inequity), and it is not clear to whether the authors consider both terms exchangable or not..

Reviewer #4

(Remarks to the Author)
The authors have addressed all of my comments. I have no further comments and support publication.

Open Access This Peer Review File is licensed under a Creative Commons Attribution 4.0 International License, which permits use, sharing, adaptation, distribution and reproduction in any medium or format, as long as you give appropriate credit to the original author(s) and the source, provide a link to the Creative Commons license, and indicate if changes were made.
In cases where reviewers are anonymous, credit should be given to 'Anonymous Referee' and the source.
The images or other third party material in this Peer Review File are included in the article’s Creative Commons license, unless indicated otherwise in a credit line to the material. If material is not included in the article’s Creative Commons license and your intended use is not permitted by statutory regulation or exceeds the permitted use, you will need to obtain permission directly from the copyright holder.
To view a copy of this license, visit https://creativecommons.org/licenses/by/4.0/

Authors' Response to Review Comments:

The authors would like to thank the four anonymous reviewers and the associate editor for their time and constructive comments regarding our manuscript. We have acknowledged the hard work of the editor and reviewers in the Acknowledgements section of the revised manuscript.

In this document, the authors list the major comments made during the review of the original manuscript and, underneath each comment, **our responses are written in bold font**.

Reviewer #1 Comments:

This manuscript presents an interesting study that connects historical public policy with current flood risk exposure. The authors analyze neighborhoods in 202 U.S. cities that were historically graded by the Home Owners' Loan Corporation and demonstrate that residents in formerly redlined (D-graded) areas experience disproportionately higher flood exposure today.

The topic of this research is highly relevant as it links historical urban development, social inequality, and environmental vulnerability. The study offers new insights for equitable flood management and urban planning by revealing the persistent impacts of discriminatory housing policies on present-day climate risks.

The analysis framework is clear and straightforward. The data sources, modeling approach, and results are easy to follow, and the conclusions are well supported by the evidence. The manuscript is well written, and it tells a compelling story without relying on complex technical methods, which makes it accessible to a broad readership.

Overall, we did not identify major flaws in the design or interpretation, and recommend the manuscript for publication in Nature Communications after the following comments are addressed.

Major comments

The study uses the Fathom-US flood model, which provides high-resolution coverage of fluvial (river-driven), pluvial (rainfall-driven), and coastal flooding across the U.S. for multiple return intervals. The results in Figure 2 clearly demonstrate that the proximity of communities to waterways plays a key role in determining flood exposure. D-graded neighborhoods experience a greater share of the flood burden in 146 cities, largely due to their closer proximity to rivers or coasts. However, it appears that pluvial flooding—which results from rainfall-generated overland flow and ponding before runoff enters watercourses or drainage systems—has not been fully represented in the analysis or discussion. Because pluvial flooding is not necessarily correlated with distance to waterways, proximity alone may not adequately capture its influence on neighborhood-level exposure. It would be valuable to clarify whether pluvial flooding contributes differently to exposure patterns across neighborhood grades. For instance, do the results suggest that pluvial flooding affects all communities more evenly, or could it also reinforce disparities in certain urban contexts?

We thank the reviewer for this feedback. You correctly point out that because Fathom-US simulates both fluvial and pluvial flooding, looking at overall inundation without disentangling proximity leaves the pluvial mechanism under-explored. To address this, we conducted a new spatial analysis partitioning the neighborhoods by their distance to major waterways: 'Near' (< 1 km) and 'Far' (> 1 km). Interestingly, we found that the statistically significant flood inequity ($f_D > f_A$) persists even in neighborhoods situated far from waterways. This finding directly answers your question: pluvial flooding does not affect all communities evenly; it heavily reinforces existing disparities. Because this far-field flooding cannot be explained by river proximity, it implicates systemic infrastructure deficits, such as inadequate stormwater drainage and greater impervious surface cover without green infrastructure (as explored in recent research by Salazar-Miranda et al., 2024), as compounding

drivers of exposure in D-graded neighborhoods. Salazar-Miranda, Arianna, et al. "Long-term effects of redlining on climate risk exposure." *Nature Cities* 1.6 (2024): 436-444.

We have added a new section to the Results titled *"Proximity to waterways does not fully explain flood inequity"* and included these findings in a new Figure 4 (see below). We have also extensively updated the Discussion to reflect this dual fluvial/pluvial hazard:

> **"To explain the inequitable relations observed, we explored a foundational flooding risk factor: proximity to waterways. While we found that D-graded neighborhoods are generally closer to large waterways ($p < 0.0005$; Figure 4a), this proximity is not a strong driver of flood inequity. Specifically, we observed no significant correlation between a city's proximity disparity (LD - LA) and its flood parity (fD - fA) (Figure S3). Further reinforcing this, flood inequity persisted whether neighborhoods were close to (≤ 1km) or far from (> 1 km) large rivers and coasts (Figure 4b). Such consistency suggests that proximity to flooding sources alone cannot explain the observed flood inequity. Because the Fathom-US model simulates both fluvial and pluvial flooding, the persistence of disparities far from waterways implies that D-graded neighborhoods are vulnerable not only to river and coastal flooding but also to pluvial flooding driven by excess surface runoff. This points to a systemic exposure that operates independently of proximity to waterways."**

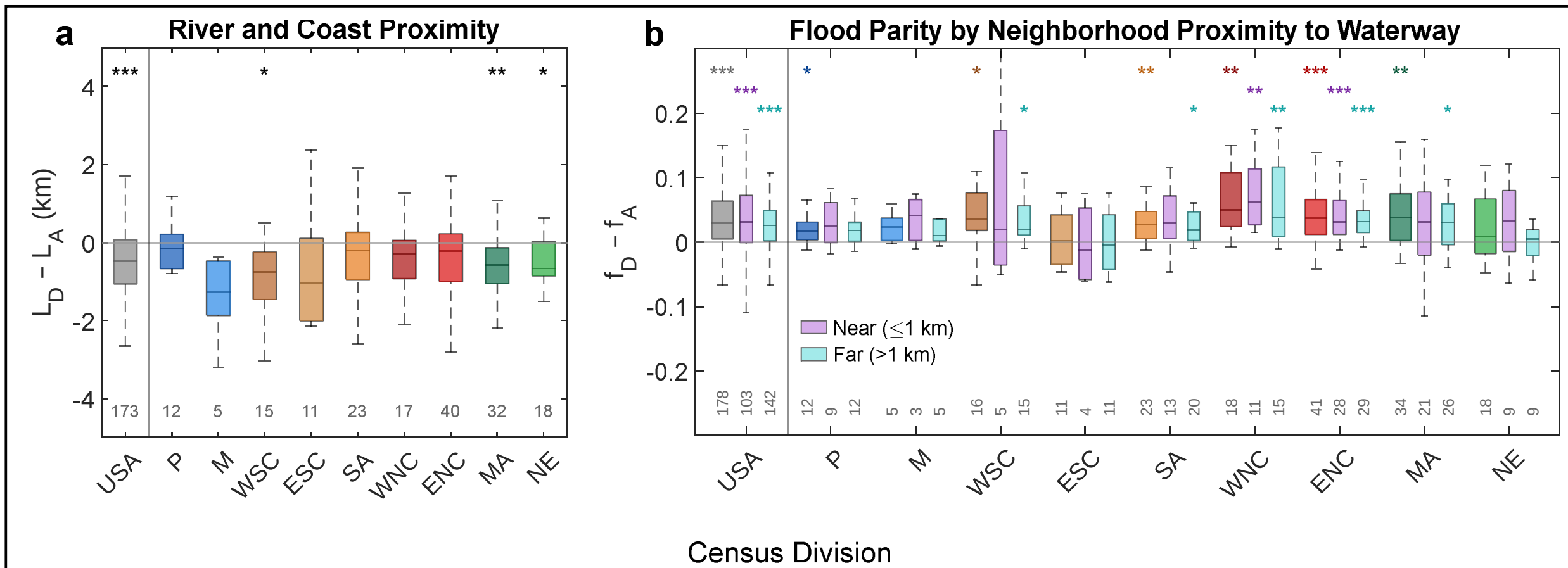

Figure 4. River and coast proximity and flood parity for the USA and Census divisions. (a) L_D and L_A represent the mean distance (in km) to the nearest river or coast of D- and A-graded neighborhoods in a city, respectively. Cities near a coast or river (stream order four or larger) were considered (n = 173). (b) Flood parity (f_D - f_A) overall and partitioned by waterway proximity. f_D and f_A represent the fraction of the flooded population to total population for D- and A-graded neighborhoods in a city, respectively. Parity is shown for all neighborhoods in eligible cities (n = 178), as well as for subsets that consider only neighborhoods near to (≤ 1 km) and far from (> 1 km) from a waterway. In all cases, fractions are shown for the 100-yr flood. The boxplots show the median, interquartile range, and whiskers. Sample sizes for each group are indicated at the bottom of the axes. Outliers are not shown for visual clarity. Asterisks denote statistical significance from Wilcoxon sign-rank tests with Benjamini–Hochberg p-value adjustments (* $p < 0.05$, ** $p < 0.005$, *** $p < 0.0005$). Division codes: Pacific (P), Mountain (M), West South Central (WSC), East South Central (ESC), South Atlantic (SA), West North Central (WNC), East North Central (ENC), Middle Atlantic (MA), and New England (NE).

Flood depth also plays an important role in determining flood damage and exposure severity. It is not clear to us how the flood extents were defined in the Fathom-US simulations used in this study. I assume a certain threshold of inundation depth (0, 0.05m or 0.1m?) was applied to classify areas as flooded. Clarifying this criterion would help readers better understand how exposure was quantified. Moreover, in cities following the Syracuse pattern, the manuscript notes that communities located near river pathways are more susceptible to flooding. If inundation depth—rather than extent alone—were integrated into the analysis, the disparity in exposure between A- and D-graded neighborhoods might become even more pronounced. Clarifying this could provide additional insight into flood risk inequality.

We thank the reviewer for suggesting this clarification. In the methods section under "Analyzing the Affected Population" we have added clarity to our inundated area methodology"

> ***"To calculate the flooded population for each MRI, we overlayed the populated neighborhood raster and the Fathom-US model products. For the purposes of this study, we consider only the extent (presence) of flooding rather than the depth at any given point. The population that resided within the area of non-zero inundation was tabulated and reported by neighborhood. The population residing within the inundation boundary for a given storm event was considered to be the 'flooded population'. The depth of flooding at any given point was not considered in this analysis."***

While we trust the FATHOM model to be as accurate as possible, we also recognize the inherent constraints of such a model to analyze localized depths at a small scale. Thus, we opted to focus exclusively on inundation patterns in this study and we have added the following sentence to our discussion to emphasize that depth is an important but complicated variable to analyze:

> ***"First, we analyzed flood extent, not depth, focusing on population impacted rather than property damage severity. While estimating severity is important, the depth-to-damage relationships that have been established exhibit considerable spatial variation and there is presently a lack of high resolution data to accurately resolve the depth, population, and redlining dynamics 1,51. Further research at a localized scale with high-resolution depth-to-damage data should be carried out to more deeply explore the broad trends found in this study."***

<u>Minor comments</u>

Line 18: What is meant by 'gap in experienced flooding'? Up to now, the abstract talks about synthetic floods (e.g. 1000-year flood) and not real floods.

We agree with the reviewer that this language was unclear, we have replaced the word "experienced" with "local" or "neighborhood" throughout the manuscript for clarity.

Similarly: Lines 201, 219, 288: "… experienced flooding …": Why experienced? To use this term, we think you should talk about actual, observed events that people have experienced. 100- and 1000-year floods are synthetic entities and not real floods.

Agreed, see above.

Lines 140-142 and Figure 2: There is a negative correlation between flood parity and river/coast proximity (in Figure 2). Have you tested the significance of this statistical relationship (fD-fA and LD-LA) directly? Would it could be interesting to plot these two variables against each other – hoping the relationship could be interpreted?

Our division-level boxplots (the old Figure 2) show that D-graded neighborhoods tend to bear relatively more of the flood impact ($f_D > f_A$) and tend to be located closer to rivers and coasts ($L_D < L_A$). The reviewer asks whether this inverse relationship with proximity and parity holds at the city-level. In the analysis below, which we put in the supplemental information (Figure S3), we show that there is not a strong direct relationship between proximity and disparity when all cities are considered together (ρ = -0.042; n = 202). When broken down by divisions, the correlation is stronger, and ranges from ρ = -0.257 in the East North Central (ENC) to ρ = 0.136 in the East South Central (ESC). Thus, while distance to waterways sets the context for flood disparities, it does not exclusively control flood disparities as other factors such as stormwater management, impervious surface, flood protection and management, etc., play an important role.

We have now added this as a major result of the paper and thank the reviewer for asking us to look deeper into the analysis, which revealed an important result.

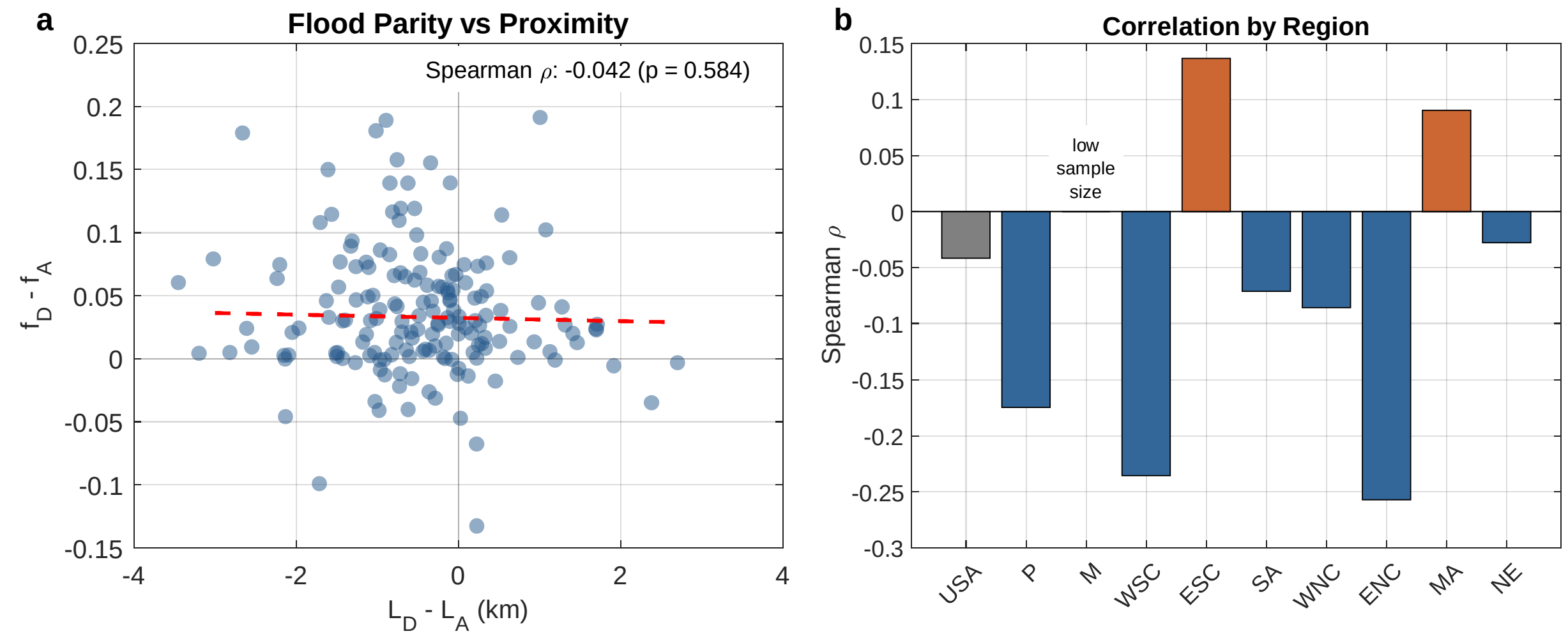

Figure S3. Correlation between flood parity and proximity disparity for the USA and Census divisions. f_D and f_A represent the fraction of the flooded population to total population for D- and A-graded neighborhoods in a city, respectively. Fractions are shown for the 100-yr flood. L_D and L_A represent the mean distance (in km) to the nearest river or coast of D- and A-graded neighborhoods in a city, respectively. Only cities meeting the criteria for both parity and proximity analyses were considered. (a) Scatter plot showing the relationship between proximity disparity ($L_D – L_A$) and flood parity ($f_D – f_A$) across all eligible cities. The red dashed line represents a robust linear fit, with the overall Spearman rank correlation coefficient (ρ) and p-value annotated. (b) Spearman rank correlation coefficients (ρ) calculated for the USA and individually for each Census division. Bar colors denote positive (orange) and negative (blue) correlations. Divisions with a sample size of $n \leq 5$ were excluded from the regional analysis due to insufficient data. Division codes: Pacific (P), Mountain (M), West South Central (WSC), East South Central (ESC), South Atlantic (SA), West North Central (WNC), East North Central (ENC), Middle Atlantic (MA), and New England (NE).

Line 204: We are unsure about the statement "… for every 1,000-year increase in MRI, an additional 3.2% [2.0%, 4.3%] of residents are exposed …". Do you mean: … for an increase from 100- to 10000-year flooding, an additional 3.2% …?

Thank you for catching this. We have clarified the text to make it clear that it refers to an increase from 100- to 1,000-yr event.

Line 237: what exactly is meant by "… navigating resources …"?

This line has been removed altogether as it ventured into an area outside of our core findings.

Line 242: The statement that D-graded neighborhoods are without adequate flood infrastructure: Is this statement supported by data or rather a (highly plausible) hypothesis? Please clarify.

This sentence has been removed to avoid confusion.

Line 280: Please explain the difference between the terms "equality" and "equity". Many readers might not be aware of it.

This line has been removed altogether as it ventured into an area outside of our core findings.

Figure S2: The title for the x-axis is missing (We assume it should be mean return interval (MRI) of flooding).

The reviewer is correct that the x-axis is the MRI. We have made that fix in the figure caption.

Reviewer #2 Comments:

I co-reviewed this manuscript with one of the reviewers who provided the listed reports. This is part of the Nature Communications initiative to facilitate training in peer review and to provide appropriate recognition for Early Career Researchers who co-review manuscripts.

We thank you for your contribution and we hope that reviewing our article was a worthwhile and informative experience for you.

Reviewer #3 Comments:

This version has undergone major revisions from several reviewers, which clearly required time and effort! I have a couple of comments here that might help finalize the manuscript:

- The abstract describes HOLC incorrectly... it was not assessing for lending as either A OR D. There is an assumption the reader is already familiar with HOLC, otherwise, this is misleading!

We have corrected the sentence in the Abstract to clarify that it was a scale:

> ***"We analyzed flood exposure across the neighborhoods of 202 US cities historically assessed for lending risk by the Home Owners' Loan Corporation on a scale from 'desirable' (A) to 'hazardous' (D)."***

- Maybe something to concern for the abstract, but line 77 has, what I think, is a critical sentence that better sets the stage for this work: "quantify the scale of flood inequity as borne by people, rather, than property..."

Constrained by the word limit of the Abstract, we opted to focus heavily on presenting the novel findings. However, we strongly emphasize the "people vs. property" distinction in the Introduction and Discussion.

- The qualifier "experienced" is used a lot to describe flooding, including the abstract... is there an un-experienced flooding? Can you eliminate to be more concise / less wording and still retain the importance of the statement?

In response to similar feedback from reviewers 1 and 2, we have replaced the word "experienced" with "neighborhood" or "local" where logical or removed the word entirely.

- I have trouble with the focus on A and D only. There is research that shows that, in coastal cities, B are better off than A because A communities are directly on the coast and their affluency affords them the ability to manage flood risk better than C and D. Alternatively, C communities can be as bad as D, if not worse (e.g., Napieralski et al., 2024). I assume the reason for A and D is to compare the two "furthest from each other" grades, but ignoring B and C may hide some relevant patterns. You also have figures that show A, B, C, and D.

We thank the reviewer for this feedback. Our initial focus on A and D was indeed to compare the furthest extremes of the scale. However, to ensure we are not hiding relevant patterns, we have introduced a new figure (Figure 2: Absolute Population Exposure) and explicitly added the B and C findings to the Abstract and Results. We highlight that C-graded neighborhoods actually bear the largest absolute flood burden, though they are proportionately represented relative to their large population size. Additionally, we have added a discussion exploring the nuances you mentioned, specifically citing Napieralski et al. (2024) regarding affluent A-graded neighborhoods and their ability to fund resilient infrastructure.

The following was added to the abstract to highlight all four HOLC grades and their major results:

> ***"This inequity follows a systematic gradient: A- and B-graded neighborhoods are underrepresented in the flooded population, C-graded neighborhoods are proportionately represented, and D-graded neighborhoods are significantly overrepresented."***

The following was added to the discussion on your recommendation of the Napierski et al., findings.

> ***"Despite modern vulnerability, formerly redlined communities often lack quality flood-protection. While overall flood susceptibility has decreased, progress remains inequitable [22]. For example, current flood management often prioritizes mitigating the potential economic impact of levee breaches, which helps to protect property but not necessarily people [41]. Disadvantaged areas with a lower tax bases thus have less capital for improved building materials and flood defense projects [16]. <u>Wealthy neighborhoods have more available capital to invest in flood prevention infrastructure and individual insurance, enabling the existence of affluent and flood-resilient communities along waterways and coastlines that would otherwise present a hazard to residents [47]</u>. This fosters a divide where affluent communities become 'risk averse' in that they have better flood defenses and longer memory of past floods, while marginalized communities are more likely to be 'risk enduring' [18]."***

See the new Figure 2 that shows the total and flooded population counts (not just fractions) of all HOLC grades:

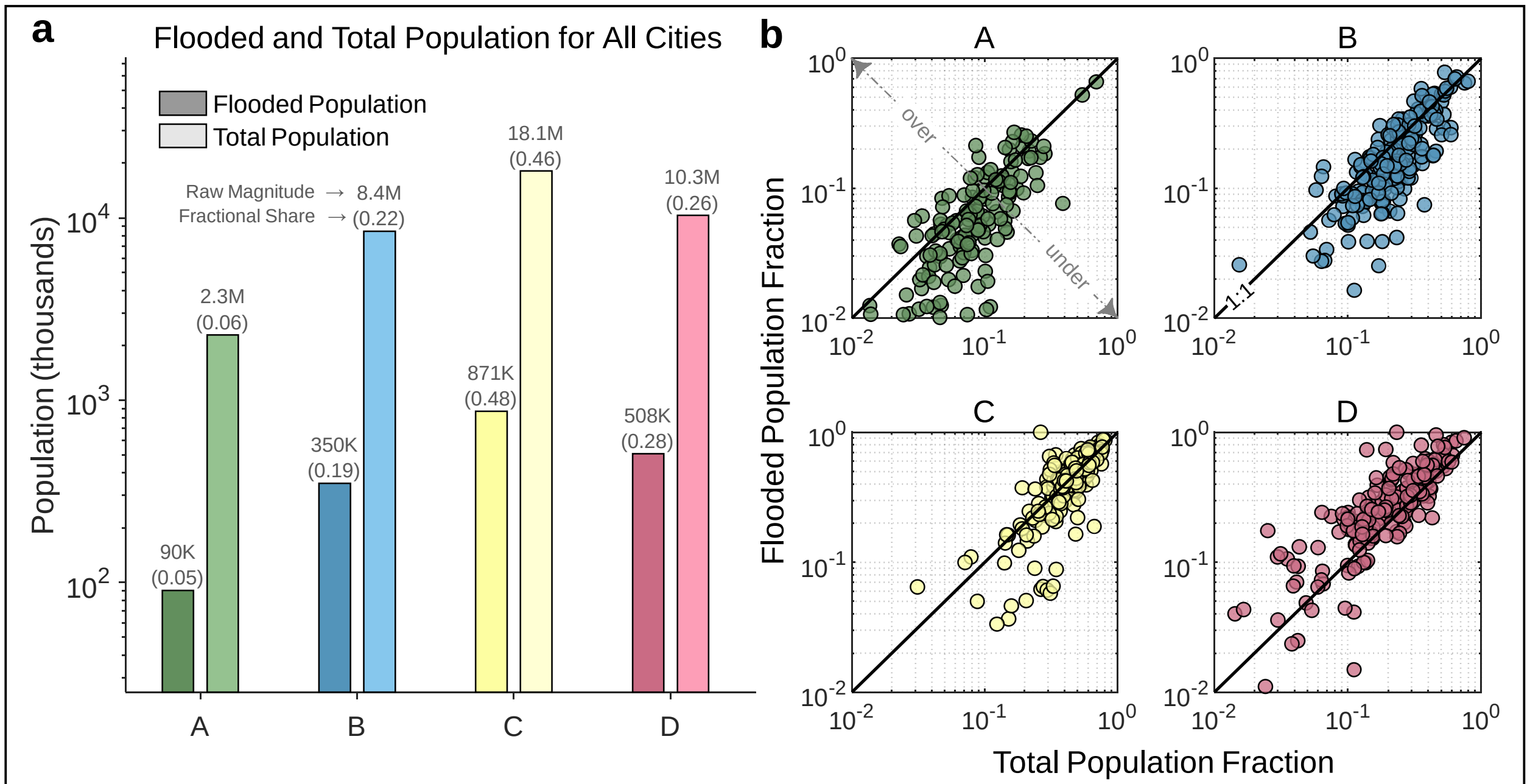

Figure 2. (a) Bars show the cumulative total population (lighter bars) and 100-year flooded population (darker bars) for all cities in the analysis by HOLC grade. Text above each bar denotes the raw population count and the fractional share of the total overall population or flooded population, respectively. (b) Markers show the 100-yr flooded population versus total population fractions for individual cities. Each panel represents a different HOLC grade. The solid black line represents a 1:1 slope, or equal share of the flood burden. Neighborhoods falling above the 1:1 line are overrepresented in flooding exposure while neighborhoods below the line are underrepresented.

- This work is solid, and adds to a growing body of work on flood inequities in a changing environment. I would say this manuscript reveals some important spatial trends and projections, but flood inequity is already well known. This includes impact of impending changes in climate, and many of those papers are not cited (e.g. Mason et al., 2017, Rickless et al., 2020, Shepherd and KC, 2015, Wing et al., 2022), nor is there a sufficient introduction to this issue (part of the "widening" of flood exposure).

We appreciate the recommended citations and have broadened our literature review to include them. Our manuscript was transferred from [REDACTED] with a more restrictive Reference list length, so we were limited to fewer references. We already had the Wing et al. reference in our manuscript, but we now also integrate the Rickless et al. and Shepherd and KC references. We were uncertain of the Mason reference given some ambiguity of the year.

Furthermore, we realized that using the word "widening" implied a longitudinal, time-series projection, which we did not model. Instead, the Fathom-US model and our methodology evaluates static MRIs to show how disparity scales with event severity. We have changed the terminology from "widens" to "amplifies" relative to event severity throughout the paper to clarify our unique contribution: showing that the systemic disparity is fundamentally worse for more extreme, low-probability events. We have made this more clear now. Further, we've changed the title of the

manuscript to remove "widening", the new title is "Disproportionate exposure to extreme floods in historically redlined communities".

- I think the citation for economic losses is outdated, as there is more up to date numbers than those from 2015! Please update with most recent estimates to be relevant.

We have updated the loss value and relevant citation to a 2025 report.

- "Flooding is driven by a combination of geologic, climatic, and hydrologic factors"... ok, but what about development, especially on floodplains or loss of floodplains, and stormwater infrastructure limitations / failures? There is a conversation in that paragraph about land use change, which is not really one of those three factors, as it is mostly controlled by human behavior. Please be clearer in statements, as the readership for this journal is quite diverse.

We have added "anthropogenic" to the list of factors in the sentence mentioned by the reviewer. Further, we have increased the depth of our discussion on the impacts of urbanization on flood risk and have added relevant citations to our discussion.

> ***"Urbanization not only concentrates people, it changes land-use patterns and often generates uniquely concentrated impervious area, aggravating flash flood conditions and increasing urban and down-stream flood risk [46]."***

And this:

> ***"Because the disparity exists whether neighborhoods are close to a major waterway or not, D-graded neighborhoods likely experience exacerbated fluvial and pluvial flooding. This finding implicates infrastructure-related factors – such as inadequate stormwater drainage, lower investment in levee protection, and greater impervious surface cover without compensating green infrastructure – as compounding drivers of flood exposure in D-graded neighborhoods [18]."***

- I would refer to Syracuse as a "case study" to prove or illustrate the concept for all 202 cities. Is that what role it plays in this manuscript? I am not sure why Syracuse was selected, especially since it can appear biased or narrow focused, as cities along a coast might show different patterns (sometimes A communities are positioned with the best views, adjacent coastlines, whereas A and B in inland cities, like Syracuse, are more distal from rivers). I just don't get the jump to Syracuse, especially since it is not mentioned previously and takes up some space that may limit depth or background elsewhere in the manuscript.

We have added clarifying language explicitly labeling Syracuse as a "case study" used to visually ground the methodology before expanding to the national statistics. We selected Syracuse because it contains all four HOLC grades, is small enough to be visualized cleanly in a single figure panel, and experiences the exact fluvial/pluvial inundation patterns that mirror the prevailing trends of the broader 202-city dataset.

We argue that the inclusion of a case study city allows the reader to visualize the patterns explored in the study, and allows them to better follow as the analysis is expanded upon. We feel the paper could not go into any further depth without this necessary context.

- Line 100: "relatively less flooding"... means what? If you use Syracuse, can you give the reader some idea as to what this means (quantify it)?

We have added the exact percentage inundation for each grade in the Syracuse case study to quantify this statement.

- Phrases like "As will be shown in the upcoming analyses" (line 114) is wordy - if eliminated, the sentence still makes sense.

We agree and have removed this and similar transition language for conciseness.

- Figure 1, showing the regions or census divisions, is an improvement from earlier version - it helps the reader understand Figure 2 better! Nice!

We thank the reviewer for the positive feedback!

- Not Even Past is mentioned a lot, but no detail is given (just mentioned in line 67-68 as a source of digitized maps). The citation is odd, maybe it is correct, but I have seen different versions for the redlining data.

We have updated these mentions to read "Not Even Past redlining database" and ensured the citation properly reflects the University of Richmond's Digital Scholarship Lab repository.

Line 140 - still discussing Syracuse as an example, as if they are the only one that is necessary to show these relationships / patterns. Again, is this true for coasts, as flooding is perhaps driven by a combination of fluvial and coastal, depending on location?

We mention Syracuse again in this line to transition into the discussion of proximity analysis. To make this clearer, we call out Syracuse as a "case study" once again. We also further explore the exceptions to the prevailing trend found in this study in the discussion (see our response to your previous comment).

- Line 161 mentions C-graded communities. Why?

In this revised section, we now analyze and report the flood burden of all HOLC-grades (A through D) to provide a complete picture of representation, as suggested in your earlier comment.

- I think the flood inequality grows with flood intensity section is important! There is a generalization of projected rainfall patterns, implying all areas will experience more intense storms. This is true for

northern states and east coast states... the others may see less precipitation but the occasional "weather whiplash" that brings the occasional heavy rain event, perhaps driven by ENSO.

We agree that discussions of global climate change impacts require more nuance. We have added text acknowledging the regional variability in precipitation trends and the lack of stationarity in flood prediction standards. We clarified that while regional trends vary, the prevailing consensus is that the frequency and severity of extreme events will increase, which makes our MRI scaling findings highly relevant.

- Line 221-222 is awkwardly written. There are a number of sentences like this that can be more direct, clear, and concise.

We have thoroughly reviewed and rewritten these sections for clarity and directness.

- Line 254: "situated"? Maybe "placed"?

Changed as advised.

- I've read through a number of articles in this journal to see writing style, and very few (if any), relied so heavily on "we" and "us". I prefer scientists limit use of first person writing when describing their work and there is too much first person writing in this manuscript.

We thank the reviewer for this feedback and understand their stylistic preference. We recognize a rift between those who prefer to write in third-person versus the first-person. We feel first-person voice allows us to use active voice and makes the paper more direct, digestible, and accessible (https://doi.org/10.1073/pnas.2316966121). We have opted to retain the active voice where helpful, but we have reviewed the manuscript to ensure it is used appropriately.

- Line 369: What is CONUS? Spell out acronym first time it is used!

We have replaced "CONUS" with "the contiguous United States."

- Line 389: Why use 2016 ACS data? Nothing more recent?

The dasymetric population map we utilized (Swanwick et al., 2022) represents the most up-to-date available spatial integration of NLCD imperviousness data and census data. Unfortunately, it was built using the 2016 ACS data. We recognize that relying on static, slightly dated data is an inherent limitation of socio-hydrologic modeling, and we have explicitly added this to our Limitations section in the Discussion.

Reviewer #4 Comments:

The paper examines differences in modern-day flood exposure across neighborhoods historically graded by the Home Owners' Loan Corporation (HOLC) in 202 U.S. cities. The authors show that neighborhoods historically designated as hazardous (D-graded) experience significantly higher flood exposure today than neighborhoods historically designated as desirable (A-graded), and that this disparity widens as floods become more severe.

Overall, the authors have satisfactorily addressed the reviewers' concerns. I have two remaining points that should be addressed before publication.

The authors acknowledge that neighborhoods graded as hazardous were historically located closer to rivers, coasts, and other flood-prone areas, and the newly added proximity analysis reinforces this point. As a result, the observed disparities in flood exposure may reflect a persistent geographic disadvantage that was later codified by HOLC grading, rather than an effect of redlining itself. I recommend making this limitation more explicit in the Discussion by clarifying that the analysis does not distinguish between flood disparities driven by pre-existing geography and those driven by redlining as a policy intervention per se.

We agree with this assessment. We have added explicit text to the Discussion to clarify this limitation:

> ***"Whether the HOLC grading system actively caused modern divestment or merely documented pre-existing spatial inequalities [30], our results demonstrate that the spatial organization of race and class codified by these maps is inextricably linked to contemporary, unequal flood exposure."***

Besides acknowledging this, the paper would benefit from at least providing some exercises trying to disentangle the causal role of HOLC from that of pre-existing geographic differences. One way to do this would be to compute the gap f_D-f_A for a sample of neighborhoods matched on geographic characteristics. By construction, this would isolate differences associated with HOLC grading from those driven by underlying geography. It would be helpful to see how the baseline estimates of disparities in f_D-f_A vary when the analysis is restricted to this more comparable sample.

Another way of saying this is that the paper tests the null that HOLC-graded areas are not differentially exposed to flooding. This null is easily rejected, which is not surprising given that HOLC areas differ systematically in geography. A more informative null would ask whether areas graded by HOLC with characteristics similar to those of non-HOLC areas are not differentially exposed to flooding. This gets to the heart of the distinction between geography and the direct effects of redlining.

We are grateful for this suggestion, as it led us to find an interesting and impactful result that we had not yet explored in our original analysis. To disentangle the geographic (fluvial) disadvantage from the infrastructure (pluvial) disadvantage, we repeated our flood parity (f_D - f_A) analysis while strictly matching/partitioning neighborhoods by their geographic proximity to waterways.

We computed the parity gap for a subset of neighborhoods located 'Near' (< 1 km) to major waterways, and another subset located 'Far' (> 1 km) from them. Our findings show that the pattern of flood inequity ($f_D > f_A$) persists regardless of distance to waterways (new Figure 4b). Because the disparity exists whether neighborhoods are close to a waterway or not, we can conclude that pre-existing geography (fluvial flooding) does not fully explain the gap. This directly implicates the built

environment—specifically inadequate stormwater infrastructure and impervious surface cover (pluvial flooding)—as a systemic driver of vulnerability in redlined areas.

We have added a new section to the Results titled *"Proximity to waterways does not fully explain flood inequity"* and included these findings in a new Figure 4 (see below). We have also extensively updated the Discussion to reflect this dual fluvial/pluvial hazard:

> **"To explain the inequitable relations observed, we explored a foundational flooding risk factor: proximity to waterways. While we found that D-graded neighborhoods are generally closer to large waterways ($p < 0.0005$; Figure 4a), this proximity is not a strong driver of flood inequity. Specifically, we observed no significant correlation between a city's proximity disparity (LD - LA) and its flood parity (fD - fA) (Figure S3). Further reinforcing this, flood inequity persisted whether neighborhoods were close to (≤ 1km) or far from (> 1 km) large rivers and coasts (Figure 4b). Such consistency suggests that proximity to flooding sources alone cannot explain the observed flood inequity. Because the Fathom-US model simulates both fluvial and pluvial flooding, the persistence of disparities far from waterways implies that D-graded neighborhoods are vulnerable not only to river and coastal flooding but also to pluvial flooding driven by excess surface runoff. This points to a systemic exposure that operates independently of proximity to waterways."**

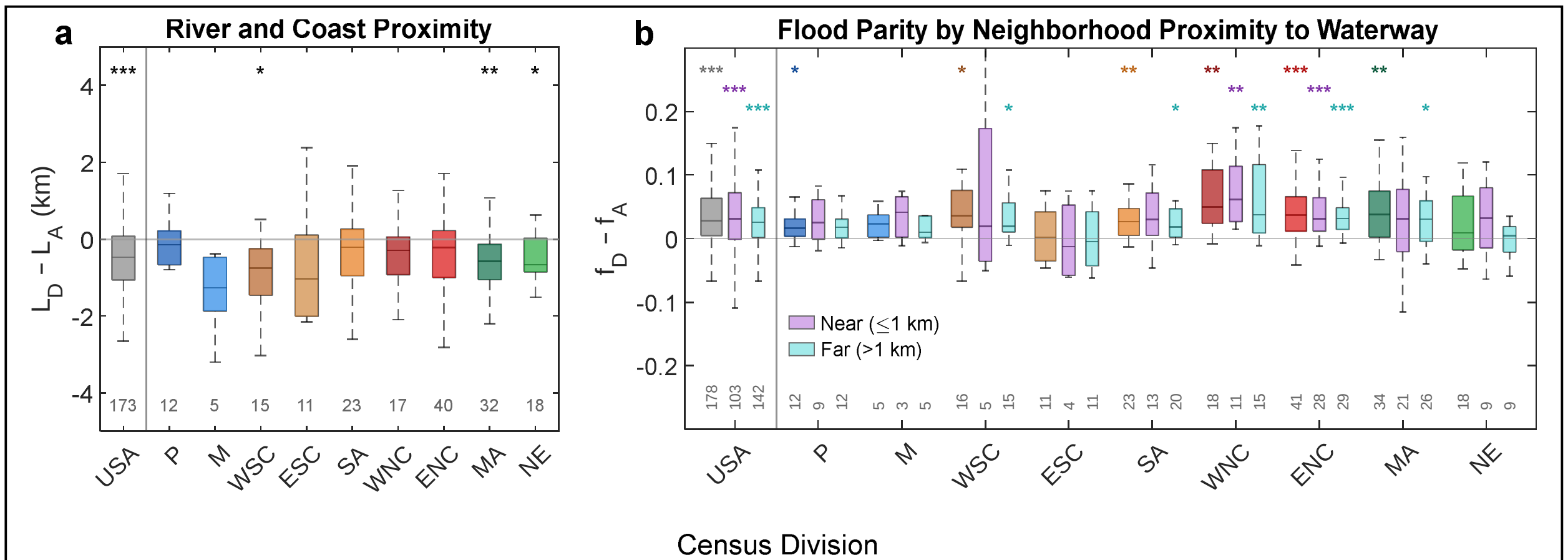

Figure 4. River and coast proximity and flood parity for the USA and Census divisions. (a) L_D and L_A represent the mean distance (in km) to the nearest river or coast of D- and A-graded neighborhoods in a city, respectively. Cities near a coast or river (stream order four or larger) were considered (n = 173). (b) Flood parity (f_D - f_A) overall and partitioned by waterway proximity. f_D and f_A represent the fraction of the flooded population to total population for D- and A-graded neighborhoods in a city, respectively. Parity is shown for all neighborhoods in eligible cities (n = 178), as well as for subsets that consider only neighborhoods near to (≤ 1 km) and far from (> 1 km) from a waterway. In all cases, fractions are shown for the 100-yr flood. The boxplots show the median, interquartile range, and whiskers. Sample sizes for each group are indicated at the bottom of the axes. Outliers are not shown for visual clarity. Asterisks denote statistical significance from Wilcoxon sign-rank tests with Benjamini–Hochberg p-value adjustments (* $p < 0.05$, ** $p < 0.005$, *** $p < 0.0005$). Division codes: Pacific (P), Mountain (M), West South Central (WSC), East South Central (ESC), South Atlantic (SA), West North Central (WNC), East North Central (ENC), Middle Atlantic (MA), and New England (NE).

Authors' Response to Review Comments:

In this document, the authors list the major comments made during the review of the original manuscript and, underneath each comment, **our responses are written in bold font**.

Reviewer #1 Comments:

The authors have responded well to all reviewer comments. I feel that the revision strengthened the already strong manuscript. I recommend publication. I have only 2 very minor issues:

(1) Line 40 and 404: Pluvial flooding and flash flooding are set equal, but there is a slight difference between these 2 types of flooding. Typical definitions are:
- Pluvial flooding refers to flooding caused by extreme rainfall overwhelming drainage systems or ground absorption, independent of rivers, often called surface water or rainfall flooding.
- Flash floods are sudden high-velocity floods from intense rain (or other triggers like dam breaks), occurring within minutes to hours (typically < 6h).

I propose that the authors only use the term pluvial flooding and delete the term flash flooding (also in line 257).

We agree with the reviewer and we have removed "flash flood" in locations where it came across as being synonymous with pluvial.

(2) Use of equity and equality: In my earlier review, I have asked to explain the difference between the terms "equality" and "equity". Often, there is a difference between these 2 terms, but probably not for everybody. The authors responded "… This line has been removed altogether as it ventured into an area outside of our core findings…" While this line has been removed, the general issue still persists. The manuscript contains 21 times the term equality (or inequality) and 9 times the term equity (or inequity), and it is not clear to whether the authors consider both terms exchangable or not.

We thank the reviewer for pressing on this distinction. The two terms carry different meanings in this literature, and we have now applied them consistently throughout the revised manuscript.

Equality/inequality refers to a quantitative, measurable state: whether two groups share flood burden in equal proportion. Every statement that reports or interprets a numerical result (e.g., a deviation from the fD = fA parity line, a binomial sign-test outcome, or a comparison of flooded fractions across HOLC grades) now uses "equality/inequality."

Equity/inequity carries a normative dimension, describing systemic imbalances rooted in historical policy and structural disadvantage that extend beyond any single metric. We retain "equity/inequity" only in the Discussion, where we interpret the broader societal significance of our findings and discuss policy implications.

In practice, this means "flood inequality" describes what we measure, and "flood inequity" describes what those measurements reflect about systemic disparities. We believe this distinction strengthens the manuscript by making clear that our empirical claims (inequality) are grounded in data, while our interpretive claims (inequity) are appropriately scoped to the Discussion.
